# Integrating evidence, models and maps to enhance Chagas disease vector surveillance

**DOI:** 10.1371/journal.pntd.0006883

**Published:** 2018-11-29

**Authors:** Alexander Gutfraind, Jennifer K. Peterson, Erica Billig Rose, Claudia Arevalo-Nieto, Justin Sheen, Gian Franco Condori-Luna, Narender Tankasala, Ricardo Castillo-Neyra, Carlos Condori-Pino, Priyanka Anand, Cesar Naquira-Velarde, Michael Z. Levy

**Affiliations:** 1 Laboratory for Mathematical Analysis of Data, Complexity and Conflicts, Division of Epidemiology and Biostatistics, University of Illinois at Chicago, Chicago, IL, United States of America; 2 Division of Hepatology, Department of Medicine, Loyola University Medical Center, Maywood, IL, United States of America; 3 Department of Biostatistics, Epidemiology & Informatics; Perelman School of Medicine, University of Pennsylvania, Philadelphia, PA, United States of America; 4 Zoonotic Disease Research Laboratory, One Health Unit, Facultad de Salud Pública y Administración, Universidad Peruana Cayetano Heredia, Lima, Perú; Imperial College London, Faculty of Medicine, School of Public Health, UNITED KINGDOM

## Abstract

**Background:**

Until recently, the Chagas disease vector, *Triatoma infestans*, was widespread in Arequipa, Perú, but as a result of a decades-long campaign in which over 70,000 houses were treated with insecticides, infestation prevalence is now greatly reduced. To monitor for *T*. *infestans* resurgence, the city is currently in a surveillance phase in which a sample of houses is selected for inspection each year. Despite extensive data from the control campaign that could be used to inform surveillance, the selection of houses to inspect is often carried out haphazardly or by convenience. Therefore, we asked, how can we enhance efforts toward preventing *T*. *infestans* resurgence by creating the opportunity for vector surveillance to be informed by data?

**Methodology/principal findings:**

To this end, we developed a mobile app that provides vector infestation risk maps generated with data from the control campaign run in a predictive model. The app is intended to enhance vector surveillance activities by giving inspectors the opportunity to incorporate the infestation risk information into their surveillance activities, but it does not dictate which houses to surveil. Therefore, a critical question becomes, will inspectors use the risk information? To answer this question, we ran a pilot study in which we compared surveillance using the app to the current practice (paper maps). We hypothesized that inspectors would use the risk information provided by the app, as measured by the frequency of higher risk houses visited, and qualitative analyses of inspector movement patterns in the field. We also compared the efficiency of both mediums to identify factors that might discourage risk information use. Over the course of ten days (five with each medium), 1,081 houses were visited using the paper maps, of which 366 (34%) were inspected, while 1,038 houses were visited using the app, with 401 (39%) inspected. Five out of eight inspectors (62.5%) visited more higher risk houses when using the app (Fisher’s exact test, p < 0.001). Among all inspectors, there was an upward shift in proportional visits to higher risk houses when using the app (Mantel-Haenszel test, common odds ratio (OR) = 2.42, 95% CI 2.00–2.92), and in a second analysis using generalized linear mixed models, app use increased the odds of visiting a higher risk house 2.73-fold (95% CI 2.24–3.32), suggesting that the risk information provided by the app was used by most inspectors. Qualitative analyses of inspector movement revealed indications of risk information use in seven out of eight (87.5%) inspectors. There was no difference between the app and paper maps in the number of houses visited (paired t-test, p = 0.67) or inspected (p = 0.17), suggesting that app use did not reduce surveillance efficiency.

**Conclusions/significance:**

Without staying vigilant to remaining and re-emerging vector foci following a vector control campaign, disease transmission eventually returns and progress achieved is reversed. Our results suggest that, when provided the opportunity, most inspectors will use risk information to direct their surveillance activities, at least over the short term. The study is an initial, but key, step toward evidence-based vector surveillance.

## Author summary

Chagas disease is a serious infection that is spread by blood-sucking insects called ‘kissing bugs.’ These bugs live in and around human homes, and until recently, they infested thousands of human homes throughout Arequipa, the second largest city in Perú. However, a decades-long control campaign drastically reduced the number of infested houses, and the city is now in a stage where health personnel annually inspect a sample of houses throughout the city for kissing bug reinfestation. A large amount of information was collected during the control campaign that could be used to help identify the houses at highest risk for re-infestation, so we developed a cell phone app to provide this information to health personnel in the form of interactive, user-friendly risk maps. We carried out a pilot study to see if health personnel would use these maps to select houses to inspect for re-infestation, and we found that most inspectors did use the information. We also observed that using the app did not slow the inspectors down, which can be an issue when introducing new technology. Our results suggest that the app could be a useful tool for monitoring diseases spread by insects in cities.

## Introduction

### Background

Chagas disease is a neglected tropical disease (NTD) endemic to the Americas with a current estimated prevalence of six to nine million people worldwide [1,2] and 70 million more at risk [3]. An estimated 30% of those with Chagas disease will develop serious cardiac and/or gastrointestinal problems for which there is no vaccine or cure [4,5]. The etiological agent of Chagas disease, *Trypanosoma cruzi*, is a parasite of mammals that is transmitted between vertebrate hosts by triatomine bugs [6], and vector control is at the core of large-scale Chagas disease control efforts [7–9].

Historically, Chagas disease was considered to be a rural problem [5,10,11] associated with homes made of rudimentary materials [12–15], the presence of domestic animals in and around the domicile [16,17], and/or in close proximity to less disturbed landscapes that serve as habitat for sylvatic mammal reservoirs of *T*. *cruzi* and *T*. *cruzi* vector foci [18,19]. Disease control efforts in the past were designed accordingly, to accommodate the characteristics of rural areas. However, Chagas disease is now known to be established in several urban settings, creating a new epidemiological challenge for prevention [20–28].

In Arequipa, Perú, with a population approaching one million people, Chagas disease is an urban problem due to widespread domestic infestation by the triatomine bug species *Triatoma infestans* [23,28–37]. In 2002, a vector control campaign targeting *T*. *infestans* was implemented in Arequipa, and today the bug is nearly eliminated from the city. The campaign is now in its 16^th^ year; over 70,000 households in 16 out of 18 target districts have been treated with insecticides in what was called the ‘attack’ phase of the campaign. These houses are now in the surveillance phase of the campaign, in which the highly challenging task of monitoring for vector resurgence is carried out through annual inspections of a fluctuating proportion of houses in each district. Although the 'attack' phase of the campaign generated a great amount of data relevant to the risk of subsequent vector infestation [30], these data are rarely used to inform the selection of houses to visit in the surveillance phase. Rather, the selection of houses is often carried out haphazardly or by convenience. Therefore, we asked, how can we harness the extensive data collected during the attack phase to enhance vector surveillance, and continue the considerable progress made toward the elimination of *T*. *infestans* from Arequipa?

### VectorPoint: Infestation risk maps to support independent decision making

To this end, we developed a cloud-based, open-source mobile app that provides vector infestation risk maps for use by health inspectors. The app, which we call ‘VectorPoint,’ is intended to enhance vector surveillance by giving inspectors the opportunity to incorporate infestation risk information into their process of selecting houses to inspect for *T*. *infestans*. Risk information is generated by a predictive model that calculates infestation risk estimates using data from the attack phase of the control campaign, in combination with new data collected during the surveillance phase. The app also provides a data entry function to collect new surveillance data. Upon collection, new data are sent directly to a virtual server, and then incorporated into the next run of the model, after which they are immediately visualized in the risk maps.

There are currently several apps for disease surveillance in resource limited settings, the most common being SMS-based apps (FrontlineSMS [38–40], RapidSMS [41,42], U-Report [43,44], Ushahidi [40,45], CycleTel [46,47], Geochat [48], among others [49]; see [50] for a thorough review of SMS apps for disease surveillance), and generic software and tool collections that offer mobile device-based data collection as their primary function, and some combination of basic data analysis, visualization and/or mapping as secondary functions (SAGES [51], Open data kit [52–54], Epicollect [54–56], eMOCHA [57,58], Medic mobile [59], Magpi [formerly Episurveyor, 60–62], DataWinners [63], and PhiCollect [64], among others [65–69]). A small number of apps have been developed for vector surveillance (Dengue Chat [70], CHAAK [71], eMOCHA [72]), with the primary features being data collection based in social networking and community based surveillance [70], and data collection using electronic forms and/or SMS [71,72].

VectorPoint is unique in that it supports independent decision making by the individual collecting the data, and it does not dictate a path to the user or mandate which houses to surveil. Rather, it provides the opportunity to integrate risk information into the inspector’s decision making process. Collaborative approaches that give control to the end-user have been shown to contribute to the sustainability of new technologies in resource-limited settings [73], and this is an important feature of VectorPoint. However, an inherent challenge with implementing a technology that supports independent front-end user decision-making is that the user can decide not to use it. As such, the potential for the app to enhance vector surveillance lies in the hands of the front-end user, and a critical question becomes, will inspectors use the information it provides?

### VectorPoint pilot

To answer this question, we carried out a pilot trial comparing surveillance using the VectorPoint app to the current practice of surveillance using paper maps. We hypothesized that inspectors would use the risk information provided by the app, as measured by the frequency of higher risk houses visited and inspected with the app compared to the paper maps. We also looked for qualitative evidence of risk information use by analyzing daily and weekly maps of the inspector’s movement patterns throughout the search zones when using the app and paper maps. Finally, we compared measures of productivity between the app and the paper maps to ensure that the app was not hindering inspector progress, which might also discourage its use.

## Methods

### Ethics statement

All health inspectors in the field study described below participated in the study under a written informed consent approved under University of Pennsylvania IRB protocol number 824603 and Universidad Peruana Cayetano Heredia IRB protocol number 66427.

### App overview

**Fig 1:**
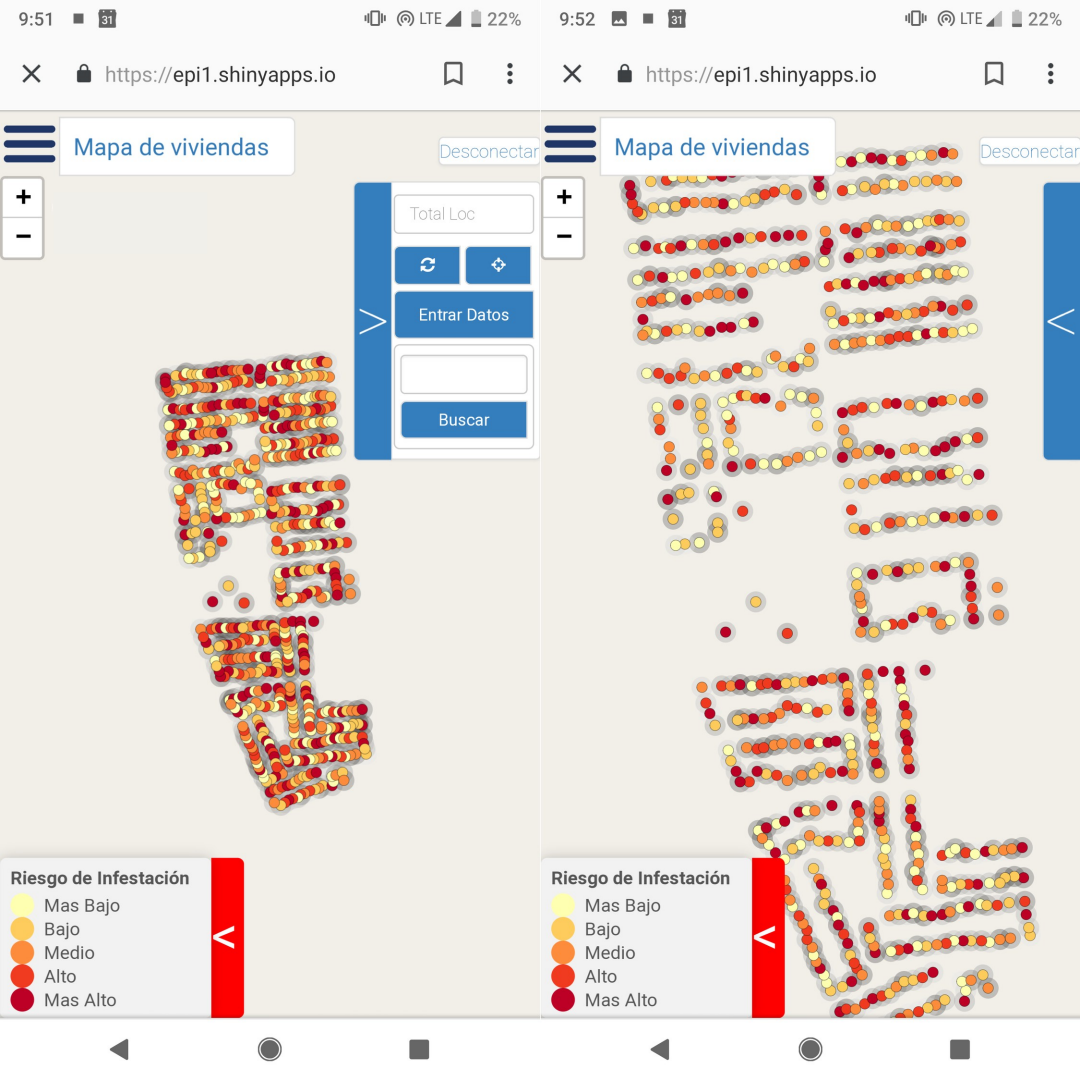
*T*. *infestans* infestation risk map zoomed out (left) and zoomed in (right). Houses are represented by dots that are colored according to their risk of infestation as estimated by the model. Legend translates to (from top to bottom): ‘Risk of infestation- Lowest; Low; Medium; High; Highest.’ Note that the images display infestation data overlay and app functions only; cartographic details (i.e., roads, parks, etc) have been removed for this publication. Images of the app with cartographic detail derived from data available at the OpenStreetMap project (openstreetmap.org) for the municipality of Arequipa, Perú, and served by MapBox (mapbox.com) are available in the VectorPoint repository, https://github.com/chirimacha/VectorPoint.

**Fig 2:**
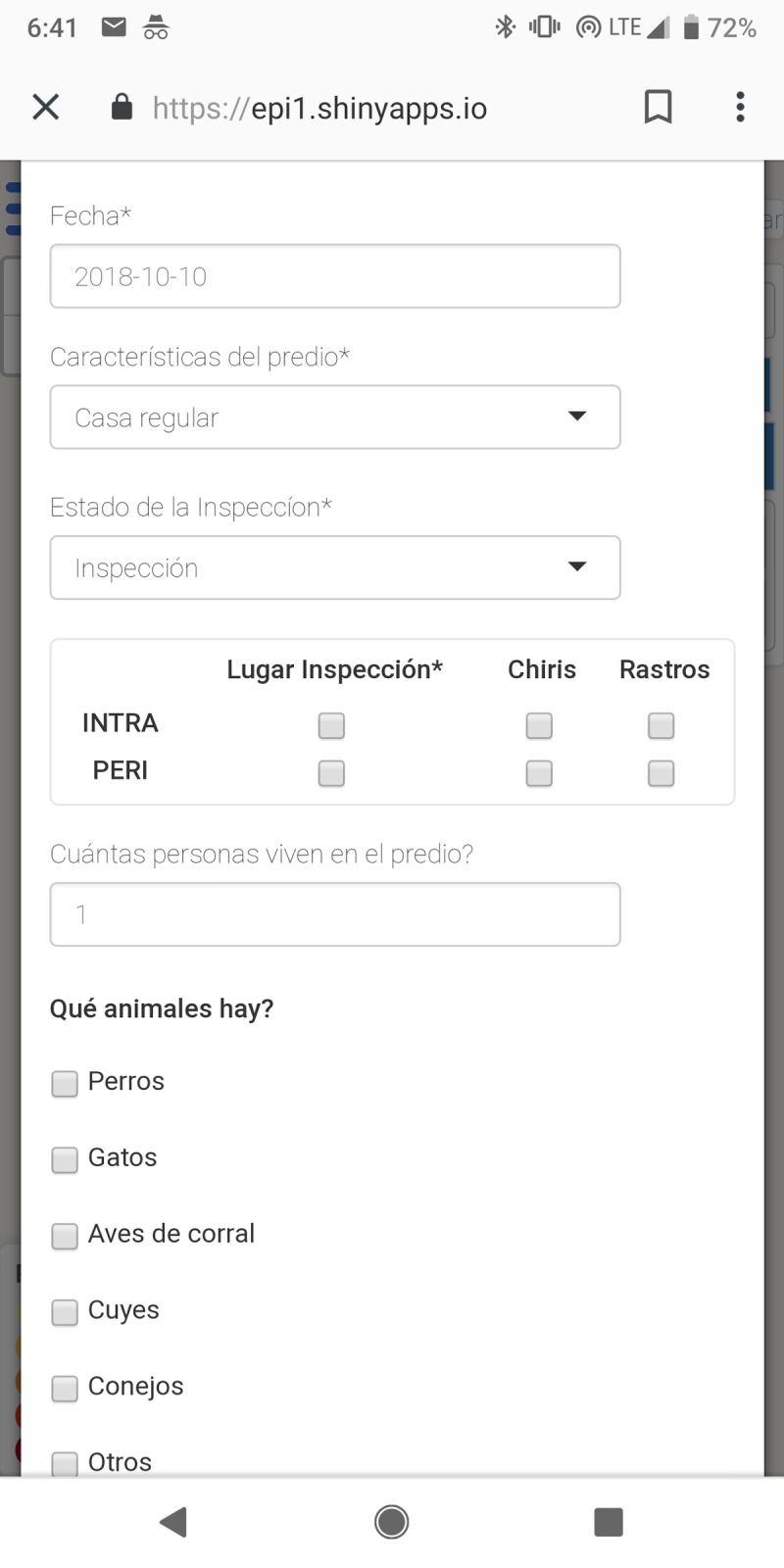
Part of the data collection form found in VectorPoint. Spanish text translates to, from top to bottom: ‘Date’; ‘Property characteristics: Regular house’; ‘State of the inspection: inspection’; ‘Inspection area: *Triatoma infestans* (‘chiris’), signs of the bugs (‘rastros’), inside the house (‘intra’), yard and patio (‘peri’)’; ‘How many people live on the property?’; ‘What animals are there?: Dogs, Cats, Poultry, Guinea Pigs, Rabbits.’

The front end (i.e., what the user sees and interacts with) of VectorPoint is a neighborhood map that displays *T*. *infestans* infestation risk at the individual house level, and a data entry tool for collecting data resulting from home inspections (Fig 1). The back end of VectorPoint (Fig 2) is composed of a spatio-temporal Gaussian field model that generates the infestation risk estimates visualized in the maps, and a relational system of cloud-based databases and servers that are used to store and send data between the predictive model and the platform that visualizes the model-generated risk estimates in the maps. Below is a more detailed description of each component of VectorPoint.

### Infestation risk map

The principal feature of VectorPoint is the risk map (Fig 1), which displays *T*. *infestans* relative risk estimates at the household level that are generated by a statistical model (detailed below). The map is intended to be used by health inspectors carrying out house-to-house *T*. *infestans* surveillance. The output from the model is presented in a simple and user-friendly format in which risk estimates are divided into five quantiles, ranging from lowest to highest infestation risk, and each quantile is then represented in the map by a color. We use the five-class, multi-hue color scheme, ‘YlOrRd,’ developed by Brewer [74] for cartography data visualization [75,76], which is color-blind friendly. The color scheme progresses from light yellow to dark red, (a color progression found to be associated with increasing hazard among Spanish speakers [77]) with saturation increasing with infestation risk, and orange representing intermediate risk. Each house is displayed in the map as a dot that is colored as one of the five colors that corresponds to the its infestation risk estimate. A legend in the corner of the map presents the colors accompanied by a one or two word description in Spanish of their corresponding infestation risk category, which translate into English as, “lowest,” “low,” “medium,” “high,” and “highest” infestation risk. Each risk category is represented in the map equally. The maps are set to display relative risk (i.e., a house’s risk of infestation relative to all other houses in the neighborhood), but they can be adjusted from the back end to display data divided into any number of quantiles, or to display absolute, instead of relative, risk estimates.

### Data collection tool

The second feature of VectorPoint is its data collection functionality (Fig 2). Inspectors can enter the data resulting from individual home visits and inspections directly into a data entry form in the app. The form is designed to collect the same data as the paper forms used by the Ministry of Health for *T*. *infestans* surveillance: date, house code (explanation below), areas of the home inspected (inside, outside, or both), number of inhabitants, number and type of domestic animals, and whether *T*. *infestans* or signs thereof (generally, eggs, feces or exuviae, grouped together as ‘rastros,’ meaning ‘traces’) were found. Radio buttons and drop-down menus are provided whenever possible for consistency, and to avoid typographical errors. After the data entry form is completed, data are encrypted and transmitted from the app to a SQL database, eliminating the step of digitizing data from paper forms.

It should be noted that *T*. *infestans* surveillance data in Arequipa are organized with four tuple identification (ID) codes assigned to each home by the Peruvian Ministry of Health at the beginning of the vector-control campaign. The four tuple consists of: province/district/locality/house. (VectorPoint is designed to be used for house to house surveillance at the locality level, which are neighborhoods ranging from 30–2000 households.) We have maintained the four tuple ID system in VectorPoint, and throughout the manuscript, we refer to the four tuple house IDs as ‘house codes.’

### Spatio-temporal model

The model in VectorPoint is designed to estimate the relative probability of *T*. *infestans* infestation of sites (primarily households) in an urban landscape. The model incorporates three types of information: (i) site covariates; (ii) the results of any previous inspections for *T*. *infestans*; and (iii) infestation history in neighboring sites. For each site, we include one covariate that is an indicator of participation in the attack phase of the vector control campaign, during which insecticide was applied to all participating households, as previous studies have shown that houses that did not participate are more likely to be infested [30]. We did not include other finer-scale risk factors for *T*. *infestans* infestation, such as guinea pig husbandry [23], because data were not available at the scale required for app.

Concretely, let the probability of vector presence, *i*, at time, *t*, be given by $\pi_{i}$. We model the probability using a logistic model with intercept, $\beta_{0}$, covariate information ${,\beta }_{1}$, and separable spatio-temporal random effects, $u_{it}andv_{it}:$

$$logit\left(\pi_{i}\right)=\beta_{0}+\beta_{1}+u_{it}+v_{it}$$

where $u_{it}\;$is a realization of the Gaussian field with a Matérn covariance structure [78,79]. The Gaussian field functions such that any adjustment to the estimate for one house affects all other houses in a given area, with a greater effect on those nearby. The Matérn function is a versatile model of covariance that includes Gaussian covariance as a special case [80]. The term $v_{it}$ is a first order autoregressive discrete time random effect.

**Fig 3:**
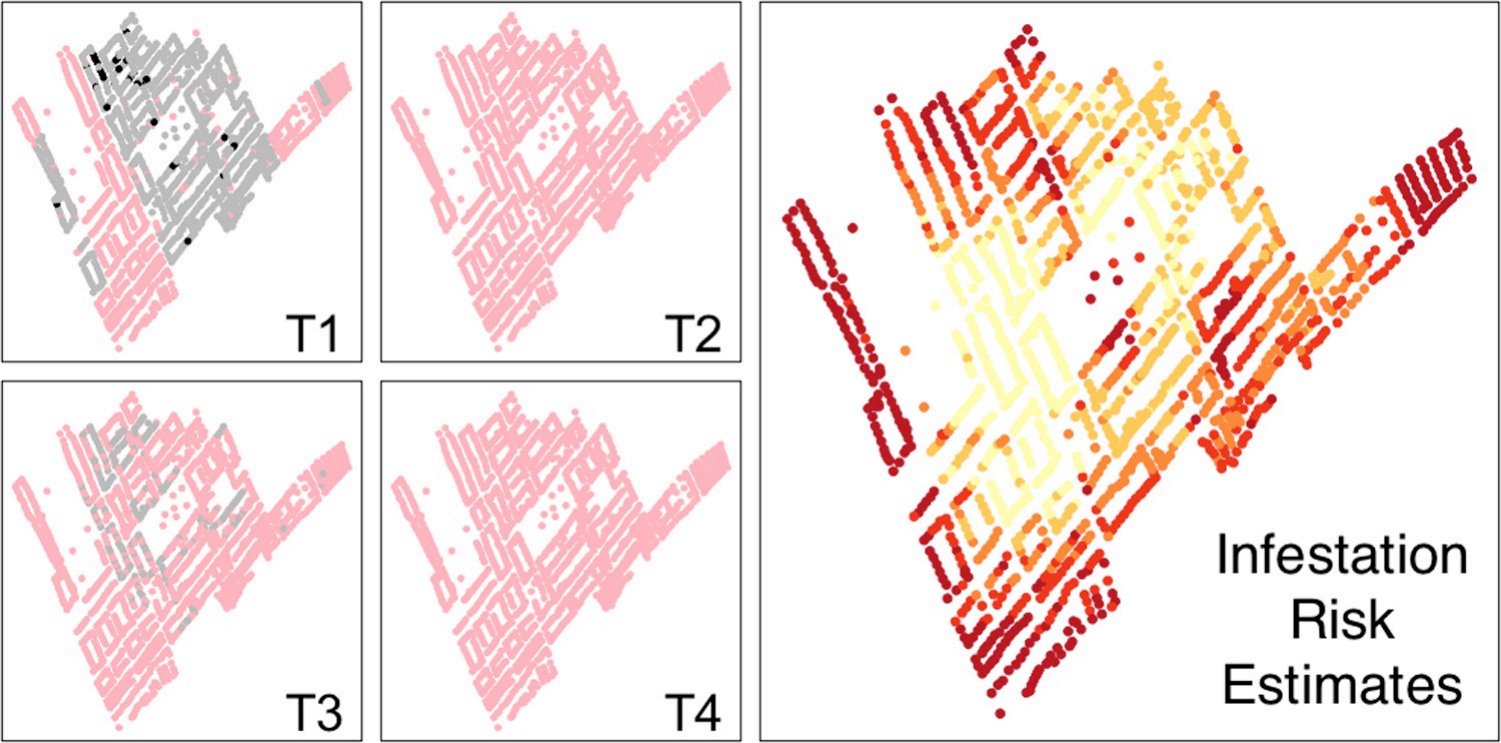
Map of a locality as represented in the model throughout the four time periods (T1-T4). Dots represent houses. T1 represents the time period of the ‘attack phase’ of the control campaign, which occurred between January 11th, 1997 and January 6th, 2014, depending on the district. In T1, grey dots represent houses that participated in the ‘attack phase’ of the control campaign but were not found to be infested with *Triatoma infestans*, and black dots represent houses that participated and were found to be infested with *T*. *infestans*. Time periods T2-T4 represent the time period of the surveillance phase (T2: January 7th, 2014—January 6th, 2016; T3: January 7th, 2016—January 4th, 2018; T4: the current calendar year, currently set to (at the time of this publication) January 5th, 2018—present). In T2-4, grey dots represent houses that were inspected and not found to be infested with *T*. *infestans* during the surveillance phase.

As mentioned earlier, the model takes into account the inspection history of each household/site. We currently include four discrete time periods (Fig 3). We selected our time periods in reference to the phase of the vector control campaign in each area. The earliest point reflects the 'attack' phase of the campaign, which occurred between January 11th, 1997 and January 6th, 2014, depending on the district. The second time period is the early surveillance period, and it includes all inspection data collected between January 7th, 2014 and January 6th, 2016. The third time period includes inspection data collected between January 7th, 2016 and January 4th, 2018. The final time point reflects the current calendar year, currently set to (at the time of this publication) January 5th, 2018—present. The later time periods can be adjusted if needed. The predicted probability of infestation for the most recent time point is visualized in the app.

We fit the model with integrated nested Laplace approximations (INLA) using the R-package, “INLA” [81,82]. To account for effects of streets as semi-permeable barriers to the spread of *T*. *infestans* [29], we used an extension of a Gaussian Field model in which we stretched the city map so the geographic center (i.e., the statistical mean of the coordinates) of each city block is at a multiple (1.5;[83]) of the true distance. We maintain the within-block structure, so only the distance between blocks is stretched [83]. We set strong priors (mean = 1.17 and standard deviation = 0.01) on the covariate of not participating in the original insecticide application campaign, based on our previous analysis of this factor [30]. We set the prior on the intercept term to correspond to an expected baseline infestation prevalence of approximately 1 in 1000, with the precision matrix set to 50. This value reflects our best estimate of *T*. *infestans* infestation prevalence in Arequipa based on recent results from both passive surveillance (i.e., reports of *T*. *infestans* infestation from community members that are later confirmed by health personnel) and active surveillance (house to house surveillance conducted by our team and the Ministry of Health).

### Data flow and platform

**Fig 4:**
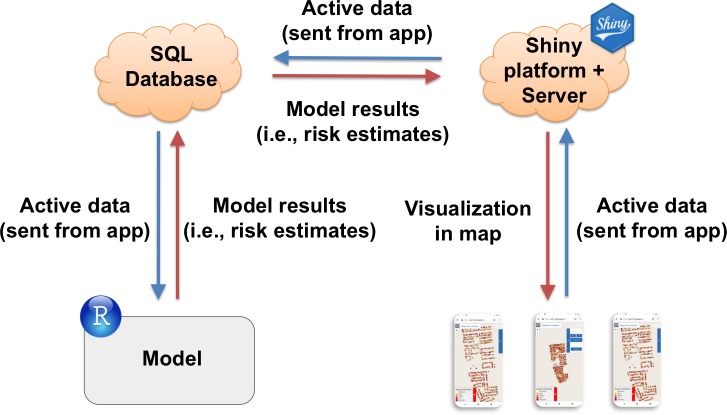
VectorPoint workflow diagram. From left, risk estimates generated by the model are first sent to an SQL database, and then sent to the Shiny platform and server for visualization in the risk map. From right, data collected by the app are sent to the Shiny server and then to the SQL database. The model then pulls the new data from the SQL database the next time it is run. All data are TLS encrypted.

Infestation risk estimates generated by the model are sent to a cloud-based database (Amazon Relational Database Service from Amazon Web Services) through the RMySQL package [84]. These data are then sent to the Shiny [85] server, which graphically renders the risk estimates in the app. Inversely, new data collected with the app are sent back to the SQL database, and incorporated into the next run of the model. We present a diagram of the VectorPoint workflow in Fig 4.

To address connectivity issues, VectorPoint has a caching function that stores partial inspection results, and retrieves them when connectivity is restored. VectorPoint supports multiple model result tables, and all operations are computationally parallel, allowing the app to be used by multiple inspectors simultaneously without speed degradation.

We constructed VectorPoint using open source software throughout to enable sharing and extensions. We built it using the Shiny package for R [85], and we implemented it entirely in the open source R programming language [82]. We mapped vector infestation predictions using the Leaflet package for R [86], and we overlaid these data on top of street data from OpenStreetMap.org. Open source code and related tools for VectorPoint can be downloaded from https://github.com/chirimacha/VectorPoint, including a link to a fully-functional installation of the app, which is available in the ‘README’ section.

### Workflow in the field

VectorPoint is located on a web server that can be accessed using a web browser on any desktop or mobile device, regardless of platform (Android, iOS, Windows, OSX, Linux, etc). Upon loading the VectorPoint web page, the inspector is presented with an authentication form in which they enter a username and password. All connections are encrypted, and risk maps can be accessed only by the study team and authorized health personnel. After user authentication, the inspector selects the locality or group of localities where they will carry out surveillance that day. The app retrieves the data for the locality from the database, and loads the corresponding risk map, zoomed out (Fig 1A). This view provides the inspector with a high-level view of the houses and their relative risk levels of infestation.

From there, the inspector zooms in on the map (Fig 1B), and selects a house to potentially visit by clicking on the corresponding dot on the map. A dialogue box will open up containing the house code, the date that the house was last visited, and whether or not the house was inspected at that time. If the inspector decides to visit that house, they can load a data entry form with the house code and date auto-filled in. If the inspector receives permission to inspect the house for *T*. *infestans*, data from the inspection is entered into the data entry form. If the inspector does not receive permission to inspect the house, it is recorded in the data entry form as one of four alternative outcomes: “interview,” “closed,” “refused,” or “return.” ‘Interview’ means that the inspector spoke with someone at the door about *T*. *infestans* infestation, but did not receive permission to enter the house and inspect it; ‘closed’ means that no one answered the door; ‘refused’ means that inspection was directly refused; and ‘return’ means that the inspector was asked to return at a later time. After each house visit, data are sent from the app to the database, regardless of visit outcome. In cases of data outages or other internet connectivity issues, inspection data can be saved and sent to the database at a later time, as described above. This process is repeated for each home visited by the inspector in a given day. At the end of the day, all data collected with the app are pulled from the database and run in the model to generate new predictions. The predictions are then pushed back to the database and visualized in the map.

### Field study design

**Fig 5:**
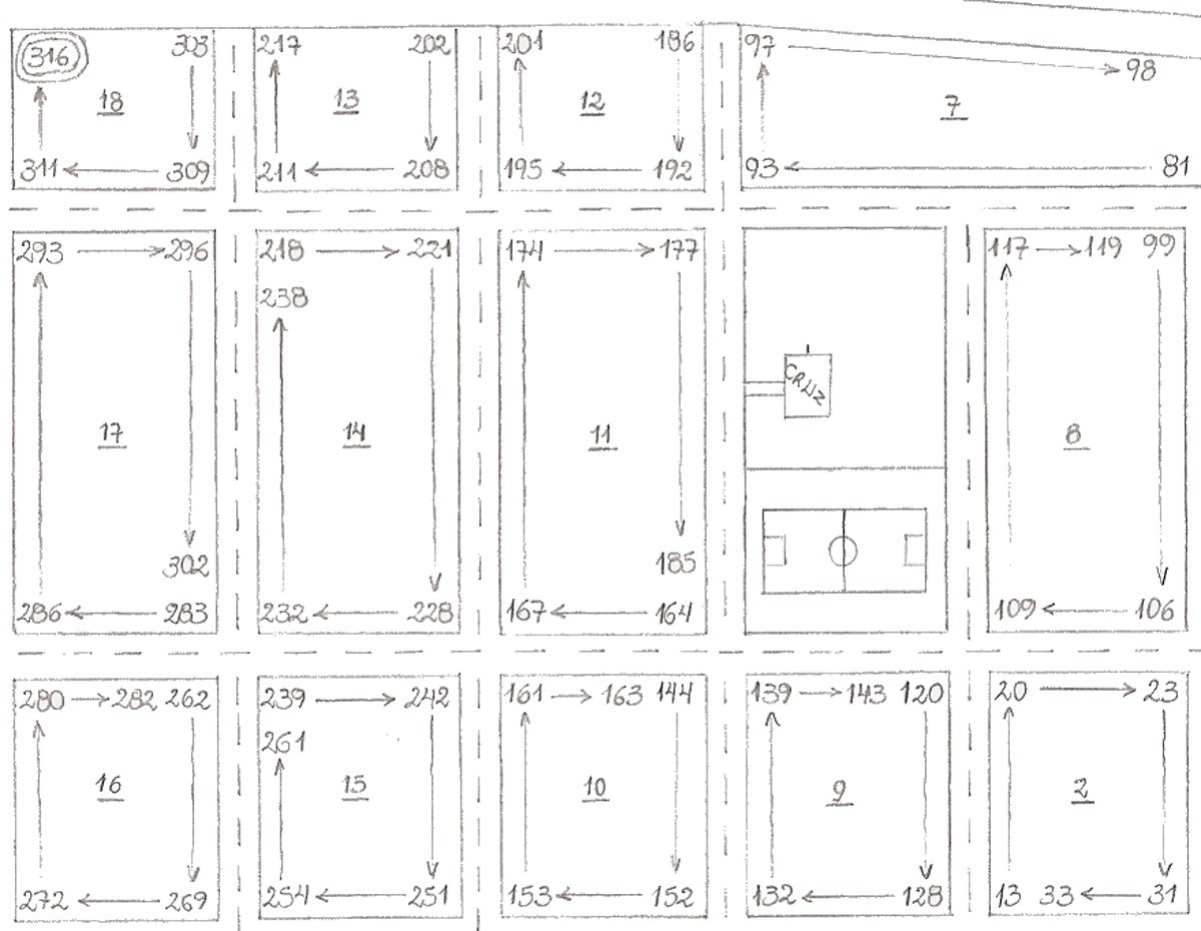
Example of a paper map used in vector surveillance under the current practice. The numbers displayed on each block are the last three digits of the house code, with arrows indicating their ascending order. For example, a block with ‘1 → 12’ indicates that houses with unicodes ending in one through 12 are on that block. Street names and other identifying information have been removed.

In the study comparing *T*. *infestans* surveillance with the app to surveillance under the current practice of using hand-drawn paper maps (Fig 5), eight members of our field team previously trained to carry out home inspections for *T*. *infestans* carried out vector surveillance in Arequipa for a total of two work weeks (10 days). At the beginning of each week, inspectors were randomly assigned (i) a zone to surveil, and (ii) if they would use the app or the paper map in that zone. Only one inspector was assigned to each search zone, which were all in the same city district. Each search zone met the following five criteria: (i) it was located in a developed area (i.e., all roads paved) within the central portion of the district, (which is safer than peripheral, less developed areas); (ii) it contained a minimum of 400 houses, and no more than 1.25 times the number of houses in the zone with the fewest houses; (iii) its area was a minimum of 0.1 km^2^, and could not be greater than twice that of the zone with the smallest area; (iv) house density was at least 2000 houses per km^2^; and (v) the search zone was in a locality where at least one house had been found positive for *T*. *infestans* during the attack phase of the control campaign. These criteria resulted in 16 search zones with 416–514 homes, areas of 0.12–0.20 km^2^, perimeters ranging between 1.62–2.37 km, and house densities ranging from 2,570 to 3,623 houses per km^2^. Half of the inspectors used the app in the first week and paper maps in the second week, and the other half used paper maps in the first week and the app in the second week. All inspectors used the same cell phone model and operating system when using the app (Samsung Galaxy J7, with Android version 7.0), to control for variation between devices. Inspectors received training in app use with these phones before starting the experiment.

Surveillance was carried out daily during normal working days (Monday-Friday) and hours (7am-1pm). Inspectors were told to carry out *T*. *infestans* surveillance as they would normally, and that their objective was simply to find *T*. *infestans*, in order to avoid perceptions that they needed to fulfill a quota of visiting a certain number of houses. The protocol for *T*. *Infestans* inspections is stipulated by the Peruvian Ministry of Health, and consists of systematic searches in all areas of the domicile and peri-domicile (pending permission by the resident), including spaces occupied by humans and live animals. Searches last approximately one person-hour; flexibility is allowed to account for the heterogeneity in the size of houses. During the search, the inspector looks for live *T*. *infestans*, in addition to *T*. *infestans* eggs, exuviae, and feces.

Inspectors using the paper maps did sometimes have access to two pieces of infestation risk information, as each house code (which is painted on the outside of the house) contains indicators of participation in the attack phase of the vector control campaign, and a '+' sign at the end of the code if the house was ever found to be infested with *T*. *infestans*. This information is available only when standing directly in front of a house looking at the house code; it is not shown in the paper maps, meaning that inspectors using the paper maps could not see the spatial distribution of houses with these risk factors. In addition, many of the house codes have been painted over by homeowners in the years since the attack phase of the campaign, so all houses with these risk indicators are not identifiable.

### Data analysis

To measure the effect of using the app in the field on inspector productivity we compared the total houses visited and total houses inspected between the app and paper maps. We also compared the proportion of total houses visited that ended up being inspected between the two mediums. We selected these metrics to test if the app was slowing the inspectors down or constricting their surveillance activities in some way, due to technical difficulties or otherwise. As mentioned above, we did not compare the number of infested houses found, since the prevalence of infestation is currently very low [29].

To investigate if inspectors used the risk information provided in the app to select houses to visit, we compared the proportion of houses visited that were higher risk houses (top two risk levels) when using the app and when using paper maps. To further investigate risk information use, we compared the proportion of total houses visited that resulted in inspection between the app and paper maps among just the higher risk houses visited (houses presented in the app as ‘highest’ and ‘high risk’) and just the lower risk houses visited (houses presented in the app as ‘medium,’ ‘low,’ and ‘lowest’ risk). We were interested in how the possession of information about a house’s estimated risk level might influence the visit outcome (i.e., if a house was inspected, closed, inspection refused, an interview took place, or the inspector was asked to return at a later time).

Finally, we looked for qualitative evidence of risk information use by comparing maps of inspector movement patterns throughout the search zones when using the app and paper maps. The daily maps for each inspector are found in the VectorPoint repository (https://github.com/chirimacha/VectorPoint). We examined movement patterns on a smaller scale, such as changes in direction and the tendency to visit every neighboring house versus skipping houses. In addition we looked at patterns on a larger scale, such as the tendency to visit all houses in one area of a zone versus visiting a few houses across several areas, as well as cumulative movement throughout the total search zone across all five days, which we refer to as ‘spatial coverage’ of the zone.

### Statistical testing

To compare the total number of houses visited and houses inspected between the app and paper maps we used a paired t-test. For analyses involving house risk level, we split the houses visited into binary categories of higher and lower risk, consisting of the top two risk levels and the bottom three risk levels, respectively. For analyses involving visit outcome, we classified visit outcomes into the binary categories, ‘inspection,’ and ‘other.’ For all metrics except total houses visited and total house inspected, we carried out a preliminary analysis using Fisher's exact test to test for differences between the app and paper maps for each individual inspector, and a Mantel-Haenszel chi-square test with continuity correction to test for an overall shift in one direction among all inspectors. We carried out a second analysis using binomial Generalized Linear Mixed Models with "Inspector ID" random intercepts to test whether the inclusion of an "app" fixed effect helped to explain any variance. In this analysis, we compared the results from a model run with only inspector ID random intercepts with results from a model run with inspector ID random intercepts plus an app fixed effect. We used the BIC scores [87] as a metric to assess if the addition of the app fixed effect improved the performance of the models, with smaller BIC scores suggesting model improvement. In addition, we evaluated the odds ratios in the app fixed effects models to understand if the app increased the odds of the outcome occurring. Models were fit by maximum likelihood (Laplace Approximation) using the ‘glmr’ function in the lme4 package [88] for R. All data analyses were carried out in the R statistical computing environment [82].

## Results

Over the course of ten days, eight inspectors visited a total of 2,119 houses, of which 767 were inspected for *T*. *infestans* (Table 1). In the five days using the paper maps, 1,081 houses were visited, resulting in 366 inspections (33.9%). In five days with the app, 1,038 houses were visited, resulting in 401 inspections (38.6%).

**Table 1:** Columns: Total number of house visits (left) and the number of house visits that resulted in inspection (right) using the app and paper maps. Rows: data for each inspector.

Inspector	Total visits		Inspections	
	*App*	*Paper maps*	*App*	*Paper maps*
A	88	90	44	37
B	184	141	65	49
C	164	175	31	19
D	111	95	81	76
E	130	123	46	43
F	126	145	47	43
G	112	114	46	56
H	123	198	41	43
Total	1,038	1,081	401	366

### Effect of the app on inspector productivity

**Fig 6:**
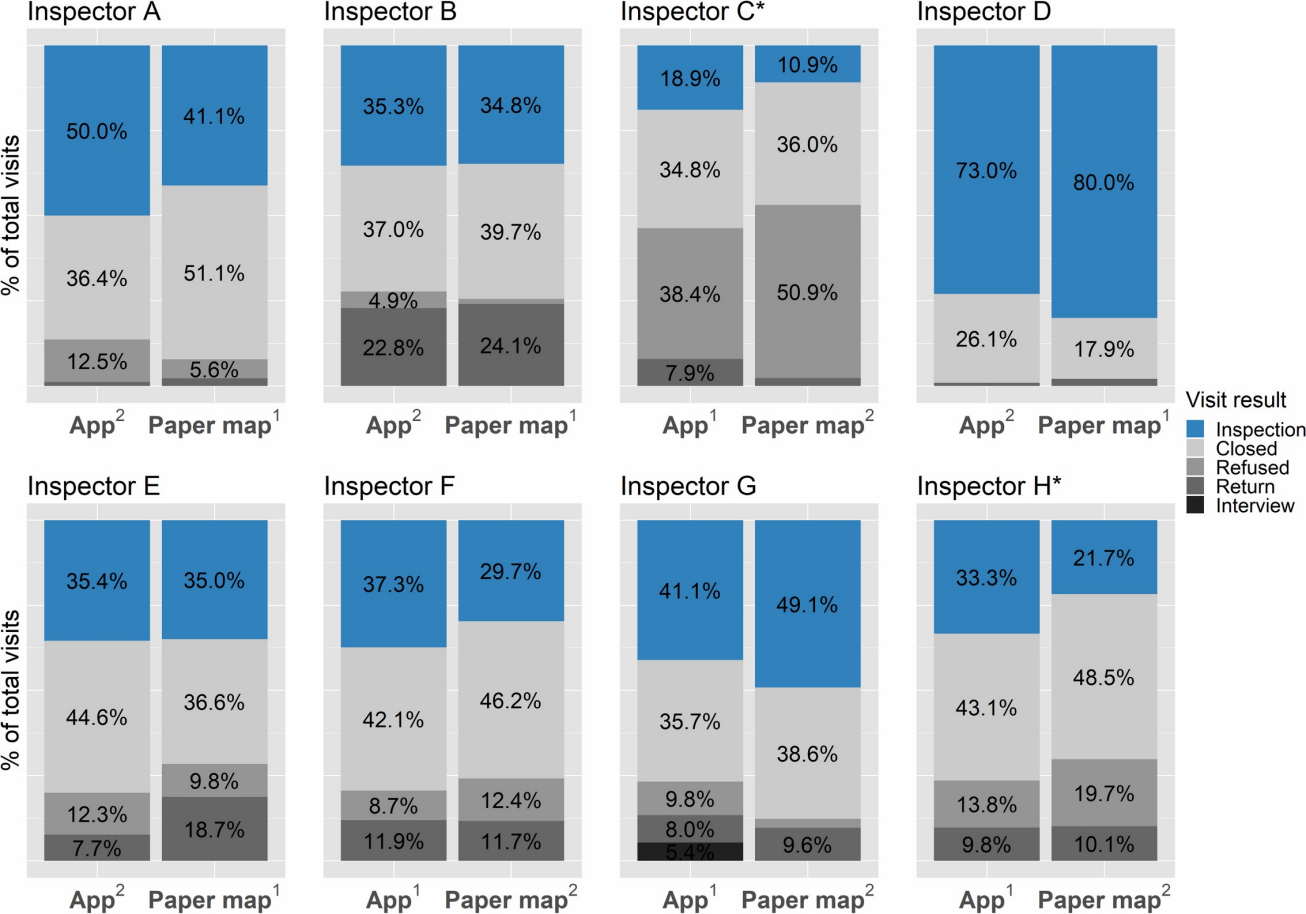
Visit outcome distributions. Inspections shown in blue, visit outcomes making up the ‘other’ category shown in shades of grey. Letters A-H refer to the corresponding inspector. *Significantly higher proportion of houses inspected when using the app (Fisher’s exact test, p<0.05).

There was no difference between the app and paper maps in the total number of houses visited (paired t-test, p = 0.67, Table 1), the total number of houses inspected (paired t-test, p = 0.17, Table 1), or the proportion of total visits that resulted in inspection (Mantel-Haenszel test, chi square = 2.63, p = 0.105, odds ratio (OR) = 1.18, 95% CI = 0.97–1.42; Fig 6), further suggesting that the app did not reduce productivity in the field. When looking at inspectors individually, no one inspected proportionally more houses with paper maps. Two inspectors, C and H, inspected a significantly higher proportion of houses when using the app as based on test p-values (Fisher’s exact test, Inspector C: p = 0.046, OR = 1.91, 95% CI = 0.99–3.76; Inspector H: p = 0.026, OR = 1.80, 95% CI = 1.05–3.08, Fig 6).

In the generalized linear mixed models (GLMMs), the odds ratio of the app fixed effect was 1.18 (95% CI = 0.97–1.42, Table 2), suggesting little effect of the app on the odds of a visited house being inspected. The standard deviation in inspector random effects was high regardless of whether or not the model included an app effect (SD = 0.76, 95% CI = 0.49–1.39 with the app; SD = 0.77, 95% CI = 0.49–1.40 without the app). The addition of the app fixed effect did not improve the model, with similar BIC scores for both models (138.71 and 138.80 for the model with and without the app, respectively). Results from the GLMMs are presented in Table 2.

**Table 2:** Results for generalized linear mixed models investigating inspector productivity and risk information use with and without the vectorpoint app as a fixed effect.

	Inspector productivity		Risk information use	
	Random inspector variation only	With app fixed effect	Random inspector variation only	With app fixed effect
**Dependent variable**	# inspections		# higher risk houses visited	
**Predictor variable**	Inspector variation	App use	Inspector variation	App use
**Inspector variation intercept** **(95% CI)**	-0.495 (-1.107–0.118)	-0.576 (-1.191–0.041)	0.571 (-0.031–1.177)	0.110 (-0.512–0.733)
**Intercept SE**	0.275	0.279	0.272	0.281
**Intercept p-value**	0.0723	0.0387	0.0356	0.697
**App effect parameter estimate** **(95% CI)**	n/a	0.163 (-0.026–0.352)	n/a	1.00 (0.808–1.202)
**App effect parameter estimate SE**	n/a	0.096	n/a	0.100
**App effect p-value**	n/a	0.0906	n/a	<2e-16
**Estimated mean probability of outcome occurring** **(likelihood profile 95% CI)**	0.379 (0.249–0.530)	0.398 (0.228–0.597)	0.639 (0.492–0.765)	0.753 (0.573–0.874)
**Random effects SD estimate** **(95% CI)**	0.767 (0.489–1.395)	0.764 (0.487–1.391)	0.756 (0.484–1.374)	0.774 (0.495–1.202)
**Odds ratio** **(95% CI)**	n/a	1.18 (0.974–1.142)	n/a	2.73 (2.240–3.321)
**BIC score**	138.80	138.71	544.30	442.90
**Model improved with app fixed effect?**	no		yes	

### Risk information use

**Fig 7:**
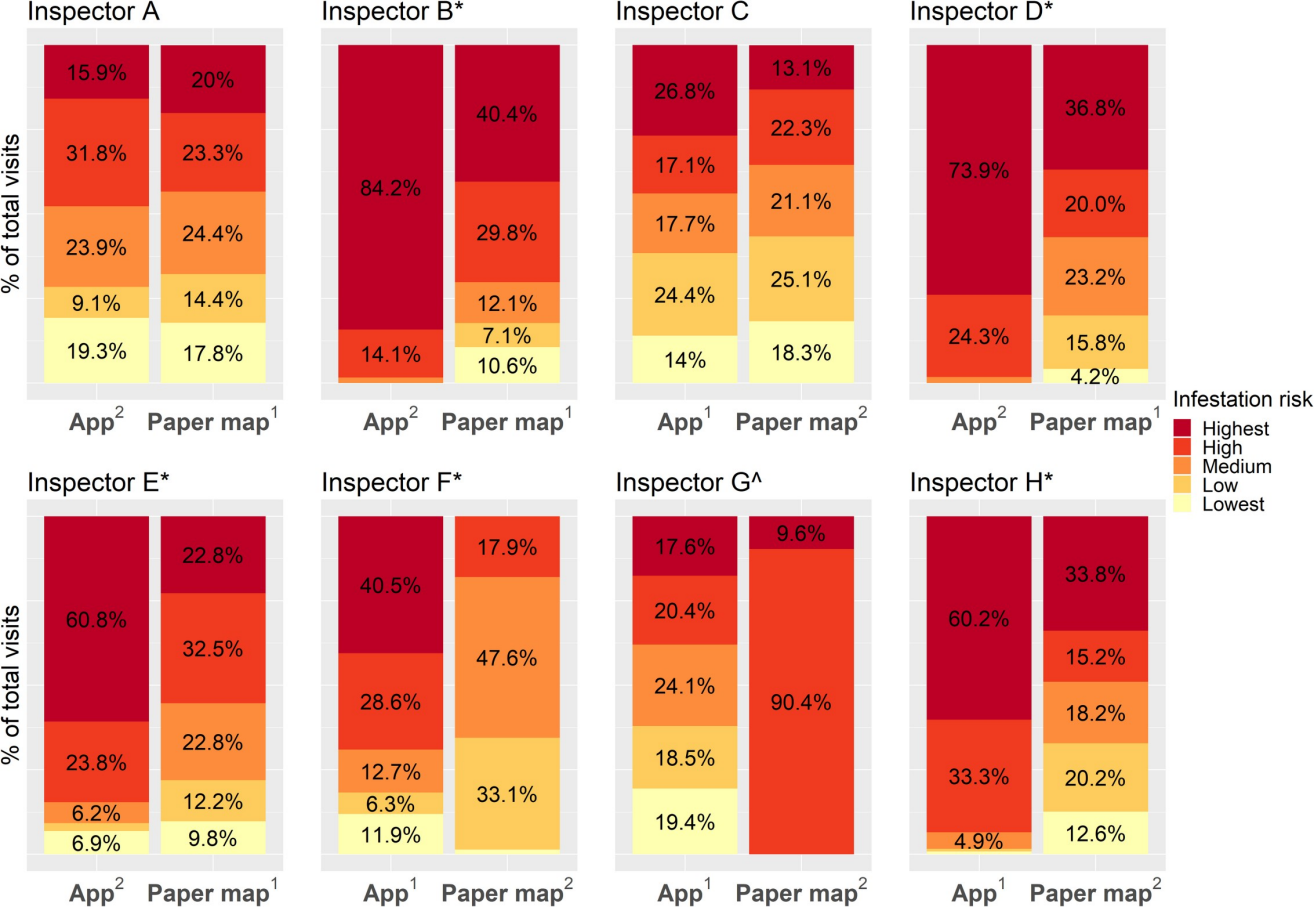
Infestation risk distribution of all houses visited for each inspector. Superscripts in the x axis text indicate arm order (i.e, which medium was used in the first week of the study, and which was used in the second week); values < 4% are not labeled due to space constraints; *p < 0.001, with more higher risk houses visited using the app; ^p < 0.001, with more higher risk houses visited using paper maps.

Five out of eight inspectors (B,D,E,F, and H; 62.5%, Fig 7), visited proportionally more higher risk houses (top two risk levels) when using the app than when using paper maps (Fisher’s exact test, Inspector B: p = 4.14e-14, OR = 25.38, 95% CI = 7.80–131.06; Inspector D: p = 1.81e-14, OR = 40.71, 95% CI = 9.94–360.00; Inspector E: p = 4.82e-07, OR = 4.42, 95% CI = 2.37–8.51; Inspector F: p < 2.2e-16, OR = 10.10, 95% CI = 5.58–18.80; Inspector H: p < 2.2e-16, OR = 14.86, 95% CI = 6.80–37.13). Two inspectors (25%), A and C, showed no difference in the proportion of total visits that were to higher risk houses when using the app (Fisher’s exact test, Inspector A: p = 0.65, OR = 1.19, 95% CI = 0.63–2.25; Inspector C: p = 0.12, OR = 1.43, 95% CI = 0.90–2.26; Fig 7), and and one inspector, G, visited proportionally more higher risk houses when using the paper map (Fisher’s exact test, p < 2.2e-16, OR = 0.0, 95% CI = 0.0–0.02; Fig 7). Overall, there was a significant shift upward in the risk level of the houses visited from the paper maps to the app (Mantel-Haenszel test, chi-square = 104.44, p < 2.2e-16, common OR = 2.42, 95% CI = 2.00–2.92; Fig 7), suggesting that inspectors did incorporate the risk information provided in the app into their selection of houses to visit.

In the GLMM, the app effect was a significant predictor of a higher risk house being visited (p < 2e-16), and it increased the odds of visiting a higher risk house by 2.73 (95% CI = 2.24–3.32, Table 2). Standard deviation in inspector ID random effects was again high in both models (0.76, 95% CI = 0.48–1.37 with the app, and 0.77, 95% CI = 0.50–1.20 without the app). BIC scores suggested that the app improved the model, with scores of 442.9 and 544.3 for the models with and without the app, respectively.

We did not observe any patterns associated with arm order (i.e, which medium was used in the first week and which was used in the second week, Fig 7). Out of the five inspectors who visited proportionally more higher risk houses using the app, two used the app first and three used the paper maps first. For the two inspectors who showed no difference between the app and paper maps, one inspector started with the app, and the other started with the paper map. The inspector who visited more higher risk houses with paper maps used the app first.

We also found no difference between the app and paper maps in the proportion of visits to higher risk houses or lower risk houses that resulted in inspection, suggesting that there is no association with knowledge of a house’s infestation risk and obtaining permission to inspect it (Fisher’s exact test, p > 0.05 for all inspectors for higher and lower risk houses; overall: Mantel-Haenszel test, higher risk houses: chi square = 1.71, p = 0.19, common OR = 1.19, 95% CI = 0.93–1.51; lower risk houses: chi square = 0.44, p = 0.51, common OR = 1.18, 95% CI = 0.78–1.78).

### Qualitative characterization of movement patterns and spatial coverage

We observed two predominant movement patterns throughout the study. The first movement pattern was more individual house focused, while the second pattern was more focused on coverage of the total search zone. (As mentioned above, daily maps for each inspector are available in the VectorPoint respository, https://github.com/chirimacha/VectorPoint.) While most inspectors consistently exhibited one pattern or the other, some inspectors displayed a mixture of both throughout the week. In the individual-house-focused movement pattern, inspectors tended to travel short distances (1–3 blocks/day), and visit every house on a block. Movement patterns appeared systematic and focused on covering all households in a small part of the zone, resulting in low spatial coverage of the total search zone. In the pattern that was more focused on search zone coverage, inspectors tended to travel longer distances, visiting a cluster of houses in one area, and then moving on to another part of the zone. Movement appeared less systematic than the individual-house-focused pattern, with a more holistic focus on the entire search zone, resulting in higher spatial coverage. There are many practical constraints in the field that will influence inspector movements, but larger movements across the zone were much more common in inspectors that tended toward the broader coverage of the search zone.

Seven out of eight inspectors (A, B,C,D,E,F and H) displayed at least one indication of risk information use in the context of the predominant movement patterns. In inspectors with individual house-focused movement patterns, risk information use was indicated when they skipped lower risk houses in between higher risk houses, or made abrupt changes in direction to avoid a cluster of lower risk houses, which sometimes resulted in increased spatial coverage of the search zone. While using the paper maps, these inspectors rarely skipped houses or changed direction, and their spatial coverage of the zone was low. Inspectors who were more focused on coverage of the total zone tended to indicate risk information use with movement that was more systematic and directed toward higher risk houses when using the app. When using the app in areas with clusters of higher risk houses, some of these inspectors adjusted their usual broader coverage movement pattern to be more individual house focused, resulting in visits to every house on a higher risk block.

We observed indications of risk information use in inspectors that did visit significantly more higher risk houses with the app (B,D,E,F,H) and in those that did not (A and C). Inspector C did not visit significantly more higher risk houses, but did skip clusters of lower risk houses on some days, suggesting they were ‘trying out’ the risk information. Inspector A greatly increased spatial coverage when using the app as compared to the paper map, which was due to a movement pattern that appeared to ‘sample’ a new risk level each day. Over the week, the inspector progressed in order of risk level from visiting lowest risk houses on day one to visiting highest risk on day five. This inspector did not visit significantly more higher risk houses using the app, but did appear to be paying attention to the risk information in the app. Indeed, by the end of the week, the inspector was skipping houses that were low and lowest risk.

Only one inspector, G, showed no qualitative or quantitative indications of using the risk information in the app to select houses to visit. In fact, this inspector unexpectedly visited significantly more higher risk houses when using the paper map, which we attribute to clustering of houses of the same risk level in the search zone. Based on examination of this inspector’s movement patterns when using the paper map, it seems that they visited more higher risk houses with the paper map because the inspector displayed a strong tendency toward fine-scale (individual house-focused) movement. They selected their day one starting point with the paper map based on house code (lowest codes first) and proceeded through the zone in numerical order. Coincidentally, the starting house was located in the beginning of a large cluster of higher risk houses, resulting in only higher risk houses being visited with the paper map.

## Discussion

Here we present a mobile app designed to provide the opportunity to incorporate data-based risk information into field surveillance for the Chagas disease vector species *Triatoma infestans* in the city of Arequipa, Perú. In our study comparing surveillance using the app to the current practice of surveillance using paper maps, we observed multiple indications that the risk information provided in the app was used in the selection of houses to inspect, suggesting that the app is a feasible tool to enhance vector surveillance and support decision making in the field.

### mHealth state of the art

mHealth tools, defined as mobile and wireless technologies for health-related objectives [89] are being introduced at a rapid-fire pace [90]. While the majority of mHealth apps are for personal uses related to aspects of individual health, the use of apps for disease surveillance in resource-limited settings has been growing steadily. The large number of data collection apps available for disease surveillance has provided the opportunity to replace often slow and cumbersome paper-based data collection systems with mobile data collection systems in which data are sent directly to a central database at the time of collection. Ideally, the shift from paper to technology will lead to increased data completeness and coverage in resource-limited settings, a critical step for achieving disease surveillance goals [91]. (Of course, new technologies bring new challenges, some of which are detailed below.) The next step is now integrating data collection functionalities with more complex tools to support ongoing decision making in the field in real or close to real time. While several data collection apps offer basic data visualization capabilities such as simple maps or bar graphs showing raw data distributions, these functionalities are often not immediately available to the individuals collecting data in the field, and the usefulness of raw data for on the ground decision making can be limited. The VectorPoint app is among the first in its marriage of data collection with predictive modeling and spatial data visualization, all of which are intended to support the individual collecting the data.

### Risk information use

As mentioned previously, for VectorPoint to meet the objective of integrating data into vector surveillance activities, inspectors with years of experience and well-established routines must adapt their decision-making processes to include at least some of the risk information in the app. Model-generated estimates are not necessarily more valuable than knowledge derived from experience, and VectorPoint is meant to complement an inspector’s experience, not replace it. In the study, we observed multiple indications that risk information was used by the inspectors. Namely, we observed a significant upward shift among all inspectors in the number of high risk houses visited when using the app compared to paper maps, and a majority of the inspectors visited significantly more higher risk houses when using the app. These results were then confirmed in a second analysis comparing generalized linear mixed models run with and without the app, in which the app was a highly significant predictor of a higher risk house being visited. In the maps of inspector movement throughout the search zones, we also observed changes in predominant movement patterns when using the app that indicated risk information use. Even inspectors who did not visit significantly more higher risk houses with the app appeared to at least ‘try out’ targeting higher risk houses or explore the different risk levels, suggesting that over time inspectors may gradually introduce the risk information into their surveillance activities.

While our results are encouraging, one inspector did not show any signs of using the risk information, and we cannot assume adoption would be universal. Indeed, our more rigorous analyses using generalized linear mixed models confirmed that random among-inspector variation is high regardless of the outcome tested. Willingness to use the information in the app may be associated with factors specific to the individual, such as age or experience with technology [89], and we may need to take into account inspector demographic information when analyzing app use in future studies in order to develop strategies for increasing risk information use. In addition, motivation to use the app may increase as more data are collected, allowing us to compare infestation indices of houses inspected while using the app versus paper maps. As mentioned above, this study was not scaled to formally compare infestation indices; only one infested house was found in the study, and the inspector was using a paper map at the time. Specialized training or more creative solutions such as incentives [92–94] or games [95,96] may be necessary in cases where inspectors have trouble engaging with the app, or if enthusiasm for the new technology wanes over time.

### Spatial coverage of surveillance zones

Although the tendency to travel or not tended to be a fixed characteristic between both arms of the study, in a few cases we observed qualitative changes in spatial coverage of the search zone when using the app. While the app is not currently designed to directly address the issue of spatial coverage, the model is expected to redistribute the relative risk estimates throughout the search zone every time it is run with new inspection data. In other words, if a house is surveyed and not found to be infested with *T*. *infestans*, infestation risk should decrease for all households within a certain distance of the uninfested house. In this way, inspectors who use the risk information in the app to target high risk houses may also display even spatial coverage of their search zone. In the study, we observed this effect only occasionally, and in some cases, inspectors targeting high risk houses displayed reduced spatial coverage of their search zone when higher risk houses were clustered. This outcome is likely due to the relatively small amount of data collected by each inspector, and the varying amounts of information available in each search area prior to the study. As detailed above, we expect risk level clustering to be dynamic, and, in the absence of a positive house, risk distribution should even out as more data are collected. The utility of the model behind the app, like all Bayesian models, increases as more data are accumulated.

Extensions to the app, or the model itself, may be useful in improving spatial coverage of surveillance, and for exploring more complex questions revolving around the benefits or drawbacks of using the app. In particular, we do not know if a trade off occurs between exploration and exploitation when using the app; by focusing on areas that are known or predicted to have a higher risk of infestation (i.e., exploitation), will we fail to detect new high risk foci (i.e., exploration)?

### Feasibility: Overcoming logistical barriers and increasing adoption

The use of mHealth tools in resource-limited settings presents inherent challenges [50,55,73] due to the financial and technical requirements of cell-phone based systems. An advantage of VectorPoint is that it uses entirely free open-source technologies, which allows large-scale deployment at little cost. The remaining operational costs involve the acquisition of mobile devices and their corresponding cellular network subscriptions. For cloud-based and web-based apps such as VectorPoint, these networks must provide data coverage of at least 2G speeds. In our field tests of VectorPoint, we found that frequent interruption and reloading of the maps were avoided when data speeds were 3G or higher. Fortunately, Peru has a robust mobile phone culture, with 117 mobile phone subscriptions per 100 people in 2016 [97], higher than the world average of 101.5. There are several networks available throughout Peru, all offering high-speed data plans. In cases where network problems do occur, VectorPoint has a caching functionality, as mentioned above, that allows data entry, although some internet connectivity is still needed for efficient use of the app. We are working towards a completely offline-capable version of VectorPoint to overcome this limitation. In our study, there was no difference in the number of houses visited between paper maps and the app, suggesting that any connectivity issues encountered did not significantly slow surveillance activities.

Being a cloud-based computing app means that VectorPoint can be run on any device without any configuration or installation, but it does require human expertise to oversee and problem-solve its backend components. Fortunately, there is a growing culture and acceptance of electronic health tools in Peru, which has been found to contribute to the sustainability of health information systems in resource-limited settings [73]. Gozzer Infante [98] reviewed 38 electronic health systems that were introduced in Peru between 2002–2010, and 66% of them were still being used in 2015. These systems include Alerta, an large multi-disease top down surveillance system [99]; Netlab, a laboratory support system for HIV treatment [100]; and Magpi, a data collection app being used by researchers to study HPV prevalence [61]. As of 2018, several mHealth apps were confirmed to be in use at the national level, (Leonardo Rojas, Peruvian Institute of Health, personal communication), such as Guardianes de la Salud [101], a mobile phone based disease surveillance system for use during the early 2018 Papal visit. In the case of VectorPoint, we collaborated with local personnel in the development of the app from the start, and they currently control both its operational use and its engineering. However, it should be mentioned that although the VectorPoint app is flexible, the predictive model must still be adapted to fit the local epidemiological and ecological characteristics of each disease surveillance scenario to which it is applied, which could present challenges in cases where expertise is limited.

### Conclusion

Our findings suggest that the VectorPoint app could be a useful tool to integrate evidence and models into epidemiological surveillance in cities. The app was designed to be used for *T*. *infestans* surveillance, but its components are cloud-based, open-source, and ready to be adapted to other appropriate scenarios, although the availability of sufficient data and/or resources may be a hurdle to overcome in some cases. VectorPoint is simple to use, but critical in function: without staying vigilant to remaining and re-emerging vector foci following a vector control campaign, disease transmission inevitably returns and progress achieved is reversed [102].

## Supporting information

S1 Dataset Study data. Data collected in the study comparing surveillance using the VectorPoint app and paper maps.
(XLSX)

S1 Translation Spanish version of manuscript. Articulo traducido al Español.
(DOCX)
