# Supplementary material for: Integrating evidence, models and maps to enhance Chagas disease vector surveillance
Source: PLoS Negl Trop Dis. 2018 Nov 29;12(11):e0006883. doi: 10.1371/journal.pntd.0006883 (PMC6289469; doi:10.1371/journal.pntd.0006883)
Supplement: S1 Translation — Articulo traducido al Español. (DOCX) [file pntd.0006883.s002.docx]

**Integración de evidencia, modelos y mapas para mejorar la vigilancia del vector de la Enfermedad de Chagas**

**Alexander Gutfraind^1,2^^, Jennifer K. Peterson^3^*^,Erica Billig Rose^3^, Claudia Arevalo- Nieto^4^, Justin Sheen^3,4^, Gian Franco Condori-Luna^4^, Narender Tankasala^1^, Ricardo Castillo-Neyra^3^,Carlos Condori-Pino^4^, Priyanka Anand^3^, Cesar Naquira-Velarde^4^, Michael Z Levy^3,4^**

**Traducido al Español por Maria Arevalo Gonzalez^5^**

**^ Ambos autores contribuyeron de manera similar**

^1^Laboratorio para el Análisis Matemático de Datos, Complejidad y Conflictos, División de Epidemiología y Bioestadística, University of Illinois at Chicago, Chicago, IL, USA
^2^ División de Hepatología, Departamento de Medicina, Loyola University Medical Center, Maywood, IL, USA

^3^Departamento de Bioestadística, Epidemiología e Informática, Perelman School of Medicine, University of Pennsylvania, Philadelphia, PA, USA

^4^ Laboratorio de Investigación Zoonótica, Unidad One Health, Facultad de Salud Pública y Administración, Universidad Peruana Cayetano Heredia, Lima, Perú

^5^Departamento deBiología, University of Pennsylvania, Philadelphia, PA, USA

***Autor correspondiente:** jenni.peterson@gmail.com; mzlevy@pennmedicine.upenn.edu

## Palabras Clave

Vigilancia entomológica, epidemiología espacial, enfermedades transmitidas por vectores, insectos triatominos, *Triatoma infestans,* Enfermedad de Chagas, Ministerio de Salud del Perú, , mapas de riesgo, ciencia de implementación

#

**Abstracto**

**Contexto**

Hasta hace poco, *Triatoma infestans,* el vector de la enfermedad de Chagas, estaba presente y difundido en la Provincia de Arequipa, Perú**;** pero gracias a los resultados de una campaña prolongada, en la cual 70,000 hogares fueron tratados con insecticidas en varias décadas, la prevalencia de viviendas infestadas es, ahora, considerablemente reducida. Para monitorear la resurgencia de *T. infestans*, la ciudad se encuentra, hoy en día, en una fase de vigilancia, en la cual, una muestra de hogares es seleccionada para ser inspeccionada cada año. A pesar de que la colección extensiva de datos de la campaña de control podría ser utilizada para informar y alterar la fase de vigilancia, la selección de estos hogares que serán inspeccionados es a menudo llevada a cabo de manera arbitraria o por conveniencia. Por lo tanto, nos preguntamos ¿Cómo podemos mejorar los esfuerzos de prevención de la resurgencia de *T. infestans* por medio de la creación de oportunidades de vigilancia basados en los datos colectados previamente?

**Metodología/ Conclusiones principales**

Con este fin, desarrollamos una aplicación móvil que provee mapas de riesgo de infestación de vectores con datos de la campaña de control, llevada a cabo en un modelo de predicción. Esta aplicación tiene el propósito de reforzar las actividades de vigilancia al facilitar la incorporación de información del riesgo de infestación en la selección de actividades de vigilancia, pero no señala los hogares que serán inspeccionados. En este caso, una pregunta crítica es si los inspectores utilizarán la información de riesgo o no. Para responder este cuestionamiento, realizamos un estudio piloto en el que comparamos la vigilancia usando la aplicación móvil en contraste con las prácticas actuales de mapas de papel. Nuestra hipótesis fue que los inspectores utilizarían la información de riesgo proveída por la aplicación, y medida por la frecuencia de visitas a hogares de alto riesgo y el análisis de los patrones de desplazamiento de los inspectores en el campo de estudio. También comparamos la eficiencia de ambas metodologías para identificar factores que podrían desincentivar el uso de la información de riesgo.

En el curso de diez días (cinco con cada metodología), 1,081 hogares fueron visitados usando los mapas de papel, de los cuales 366 (34%) fueron inspeccionados, mientras que 1,038 hogares fueron visitados usando la aplicación móvil con 401 (39%) inspeccionados. Cinco de los ocho inspectores (62.5%) visitaron más hogares de alto riesgo cuando utilizaron la aplicación (prueba exacta de Fisher, p = 0.001). Entre todos los inspectores, encontramos un cambio ascendente en la proporción de visitas a hogares de alto riesgo cuando usaron la aplicación (prueba Mantel-Haenzel, probabilidad relativa (OR=2.42, 95% CI: 2.00-2.92). En un análisis secundario usando modelos lineares generalizados, el uso de la aplicación aumentó la posibilidad de visitar un hogar de alto riesgo por un factor de 2.73 (95% CI: 2.24-3.32), lo cual sugiere que la información de riesgo proveída por la aplicación fue utilizada por la mayoría de los inspectores. El análisis cualitativo del desplazamiento de inspectores reveló el uso de la información de riesgo en siete de los ocho inspectores (87.5%). No hubo diferencias en el número de hogares visitados entre el uso de la aplicación y el de los mapas físicos (prueba t pareada, p = 0.67) o en el número de hogares inspeccionados (p = 0.17), lo cual sugiere que el uso de la aplicación no disminuyó la eficiencia de la vigilancia.

**Conclusiones/Importancia**

Al no estar vigilantes, en cuanto a focos de vectores residuales y re-emergentes, luego de una campaña de control de vectores, la transmisión de la enfermedad de Chagas puede volver con el tiempo, y el progreso conseguido se puede revertir. Nuestros resultados sugieren que cuando se les da la oportunidad, la mayoría de inspectores usarán la información de riesgo para dirigir sus actividades de vigilancia, por lo menos a corto plazo. Este estudio es un paso inicial, pero clave hacia el uso de vigilancia de vectores basada en evidencia previa.

**Resumen del autor**

La enfermedad de Chagas en una infección seria propagada por insectos hematófagos conocidos como triatominos. Estos insectos viven en hogares humanos y áreas contiguas a estos, y hasta hace poco, infestaron miles de hogares humanos de Arequipa, la segunda ciudad más grande de Perú. Sin embargo, una campaña de control que duró varias décadas redujo el número de hogares infestados drásticamente, y la ciudad se encuentra actualmente en una etapa de vigilancia anual, en la cual el personal de salud inspecciona una muestra de hogares a lo largo de la ciudad en busca del retorno de infestaciones por triatominos. Durante la campaña de control llevada a cabo en la ciudad**,** se colectaron numerosas informaciones, las cuales podrían ser utilizadas para ayudar a identificar los hogares que tienen los riesgos más altos de re-infestación; es por ello que desarrollamos una aplicación para celulares que provee esta información al personal de salud de una manera interactiva por medio de mapas de riesgo fáciles de usar. Llevamos a cabo un estudio piloto para identificar si el personal de salud usaría estos mapas en su selección de hogares para inspeccionar, y encontramos que la mayoría de inspectores utilizaron esta información. También observamos que el uso de la aplicación no disminuyó la eficiencia y velocidad de las inspecciones, algo que podría presentarse cuando se introduce una nueva tecnología en estrategias como esta. Nuestros resultados sugieren que la aplicación podría ser una herramienta útil en la supervisión de enfermedades transmitidas por vectores en ciudades.

**Introducción**

**Contexto**

La Enfermedad de Chagas es una enfermedad tropical desatendida (ETD) endémica a las Américas con una prevalencia actual estimada de seis a nueve millones de personas a nivel global [[1,2]](https://paperpile.com/c/0nGvWM/RgPTC+P21hI) y setenta millones más en riesgo [[3]](https://paperpile.com/c/0nGvWM/7QbAT). Aproximadamente el 30% de aquellos con la enfermedad de Chagas desarrollarán problemas serios del sistema cardiovascular y/o el sistema gastrointestinal para los cuáles no hay vacunas o curas [[4,5]](https://paperpile.com/c/0nGvWM/gVDEp+jLUWW). El agente etiológico de la enfermedad de Chagas, *Trypanosoma cruzi,* es un parásito de mamíferos que es transmitido entre huéspedes vertebrados por los insectos triatominos [[6]](https://paperpile.com/c/0nGvWM/wqJZ7), lo que hace el control de vectores uno de los esfuerzos fundamentales en el control a gran escala de la enfermedad de Chagas [[7–9]](https://paperpile.com/c/0nGvWM/xicFn+AGAFu+4fhlN).

Históricamente, la enfermedad de Chagas fue considerada un problema rural [5,10,11] asociado con los hogares construidos con materiales rudimentarios [12-15], la presencia de animales domésticos en los hogares y áreas contiguas a ellos [16,17], y/o la proximidad a paisajes menos perturbados por actividad humana, los cuales sirven como hábitat de mamíferos selváticos que son reservorios de *T. cruzi* y los focos de vectores de *T. cruzi* [[18,19]](https://paperpile.com/c/0nGvWM/bGUs3+K5seJ). Los esfuerzos de control previos fueron diseñados de tal manera que se acomodaron a las características de áreas rurales. Sin embargo, actualmente es conocido que la enfermedad de Chagas es establecida en varios ambientes urbanos, creando un nuevo desafío epidemiológico para su prevención [[20–28]](https://paperpile.com/c/0nGvWM/N6Wm2+FgM2T+ZSXhH+d3jOm+UXSGo+1aaKU+bltAg+9DVhs+4p641).

En Arequipa, Perú, que tiene una población de aproximadamente un millón de personas, la enfermedad de Chagas se ha convertido en un problema urbano debido a la infestación doméstica generalizada por el insecto triatomino de la especie *Triatoma infestans* [[23,28–37]](https://paperpile.com/c/0nGvWM/d3jOm+4p641+3vSSI+TFDeU+idTlt+XweAo+dj7A2+H9cuM+u4Vcx+HZol7+NwOSK). En el 2002, una campaña de control de vectores enfocada en *T. infestans* fue implementada en Arequipa, y hoy en día**,** el insecto ha sido eliminado casi del todo de esta ciudad. La campaña se encuentra en su año dieciséis con más de 70,000 hogares en 16 de 18 distritos que han sido tratados con insecticidas en la fase conocida como la fase de “ataque” de la campaña. Estos hogares ahora se encuentran en la fase de vigilancia de la campaña, en la cual la complicada tarea de monitorear la resurgencia de vectores es llevada a cabo por medio de inspecciones anuales a un número variable de hogares en cada distrito. A pesar de que la fase de “ataque” generó abundante información relevante para el riesgo de subsecuentes infestaciones de vectores [[30]](https://paperpile.com/c/0nGvWM/TFDeU), estos datos son raramente utilizados para informar la selección de los hogares que serán visitados en la fase de vigilancia. Por el contrario, estas inspecciones son llevadas a cabo de manera aleatoria y por conveniencia. Es por ello que nos preguntamos, ¿Cómo podemos aprovechar esta información extensa colectada en la fase de ataque para mejorar la vigilancia de vectores, y continuar el progreso considerable que se ha obtenido en la eliminación de *T. infestans* en Arequipa?

**VectorPoint: Mapas de riesgo de infestación para apoyar el proceso de toma de decisiones independientes**

Con este propósito, desarrollamos una aplicación basada en la nube y de fuente abierta para dispositivos móviles que provee mapas de riesgo de infestación de vectores para los inspectores de salud. Esta aplicación, llamada ´Vector Point,´ tiene el propósito de mejorar la vigilancia de vectores al proveer a los inspectores la oportunidad de incorporar información de riesgo en el proceso de selección de hogares para inspeccionar por *T. infestans.* La información de riesgo es generada por un modelo predictivo que calcula estimados de riesgo de infestación con el uso de información de la fase de ataque de la campaña de control, en combinación con nuevos datos colectados durante la fase de vigilancia. Esta aplicación también provee la oportunidad de añadir datos para recolectar nueva información durante la vigilancia. Estos nuevos datos son mandados a un servidor virtual y luego son incorporados en la siguiente ronda del modelo, lo cual permite que sean visualizados inmediatamente en los mapas de riesgo.

Hoy en día hay varias aplicaciones para la vigilancia de enfermedades en áreas de recursos limitados, de las cuales las más comunes son basadas en SMS comunicaciones (FrontlineSMS [[38–40]](https://paperpile.com/c/0nGvWM/ql1VT+ppdne+12o7Q), RapidSMS [[41,42]](https://paperpile.com/c/0nGvWM/NiJIx+QSqUS), U-Report [[43,44]](https://paperpile.com/c/0nGvWM/6f4Yu+qXNuZ), Ushahidi [[40,45]](https://paperpile.com/c/0nGvWM/12o7Q+xnbqG), CycleTel [[46,47]](https://paperpile.com/c/0nGvWM/BWgAR+R7pYk), Geochat [[48]](https://paperpile.com/c/0nGvWM/kO79c), entre otras[[49]](https://paperpile.com/c/0nGvWM/3OIRz); vea [[50]](https://paperpile.com/c/0nGvWM/MjU32) para una reseña completa de aplicaciones SMS para vigilancia de enfermedades), y software genérico y colecciones de herramientas que ofrecen la colección de datos basados en dispositivos móviles como su función primaria, y algunas combinaciones de análisis básico de datos, con visualización y/o mapeo como funciones secundarias (SAGES [[51]](https://paperpile.com/c/0nGvWM/o5zRj), kit de datos abiertos[[52–54]](https://paperpile.com/c/0nGvWM/tv60A+6hrJa+CzFtI), Epicollect [[54–56]](https://paperpile.com/c/0nGvWM/Fyam7+p0u8z+CzFtI), eMOCHA [[57,58]](https://paperpile.com/c/0nGvWM/f3SAl+QjatP), Medic mobile [[59]](https://paperpile.com/c/0nGvWM/eX2Nb), Magpi [previamente Episurveyor, [60–62]](https://paperpile.com/c/0nGvWM/EZRXn+zDzOG+nNkhK), DataWinners [[63]](https://paperpile.com/c/0nGvWM/dQccU), y PhiCollect [[64]](https://paperpile.com/c/0nGvWM/APF2k), entre otros[[65–69]](https://paperpile.com/c/0nGvWM/6AdkD+LByHa+apG8P+b0pql+MlHvF)). Un pequeño número de aplicaciones han sido creadas para la vigilancia de vectores (Dengue Chat [[70]](https://paperpile.com/c/0nGvWM/IoEum), CHAAK [[71]](https://paperpile.com/c/0nGvWM/h78UB), eMOCHA [[72]](https://paperpile.com/c/0nGvWM/jpXHQ)), con funciones primarias de colección de datos basados en redes sociales y vigilancia con base comunitaria [[70]](https://paperpile.com/c/0nGvWM/IoEum) y colección de datos utilizando formularios electrónicos y/o SMS[[71,72]](https://paperpile.com/c/0nGvWM/h78UB+jpXHQ).

VectorPoint es único ya que contiene la toma de datos independientes por parte del individuo recolectando información, y no señala un camino a tomar por el usuario o determina cuáles hogares el individuo tiene que visitar. En vez de esto, ofrece la oportunidad de integrar la información de riesgo en el proceso de toma de decisiones del inspector. Las alternativas colaborativas que le dan el control al usuario han sido reconocidas por su contribución a la sostenibilidad de nuevas tecnologías e circunstancias con recursos limitados [[73]](https://paperpile.com/c/0nGvWM/mhNJa), lo cual es una función importante de VectorPoint. Sin embargo, un desafío inherente al implementar una tecnología que apoya la decisión independiente del usuario es que el usuario puede decidir no usarlo. Siendo así, el potencial de la aplicación para mejorar la vigilancia de los vectores depende del usuario, y resulta en la pregunta de si los inspectores usarán esta información que les es provista.

**VectorPoint Piloto**

Para responder esta pregunta, llevamos a cabo un ensayo piloto para comparar la vigilancia usando VectorPoint con las prácticas actuales de vigilancia usando los mapas de papel de los distritos correspondientes. Especulamos que los inspectores utilizarían la información de riesgo proveída por la aplicación, medida por la frecuencia de los hogares en alto riesgo visitados e inspeccionados con la aplicación en comparación con el uso de mapas de papel. También buscamos evidencia cualitativa del uso de información de riesgo por medio del análisis de los patrones de desplazamientos del inspector de manera diaria y semanal en las zonas de búsqueda cuando se usaban los mapas y cuando se usaba la aplicación. Finalmente, comparamos las medidas de productividad entre la aplicación y los mapas físicos para asegurarnos de que la aplicación no estaba obstaculizando el progreso del inspector, lo cual también podría llevar a la disuasión del uso de la aplicación.

**Métodos**

**Declaración de ética**

Todos los participantes en el estudio de campo participaron consensualmente dado por un acuerdo escrito aprobado bajo el número de protocolo de IRB 824603 de University of Pennsylvania, y el número de protocolo 66427 de la Universidad Peruana Cayetano Heredia.

**Descripción de la Aplicación**

La interface frontal (i.e., con lo que el usuario interactúa) de Vector Point es un mapa del barrio que muestra el riesgo de infestación por *T. infestans* a nivel de los hogares individuales, y una herramienta de entrada de datos para los resultados de las inspecciones de hogares (Figura 1). La interface posterior de Vector Point (Figura 2) está compuesta por un modelo espacio-temporal de campo Gaussiano que genera estimaciones de riesgo de infestación visualizados en mapas, y un sistema relacional de bases de datos y servidores basados en la nube que son utilizados para guardar y enviar datos entre el modelo predictivo y la plataforma que visualiza el riesgo generado por el modelo en los mapas. A continuación está una descripción detallada de cada componente de Vector Point.

**Figura 1. Mapa de riesgo de infestación por *T. infestans* disminuido (izquierda) y aumentado(derecha). Los hogares son representados por los puntos de color, clasificados por color de acuerdo con su riesgo de infestación, tal como estimado por el modelo. La leyenda dice (de arriba abajo): ‘Riesgo de infestación- Más bajo; Bajo; Medio; Alto; Más Alto.’ Las calles en el mapa fueron extraídas de datos disponibles en el proyecto OpenStreetMap (openstreetmap.org) para la municipalidad de Arequipa, Perú, y ubicadas en servidores de MapBox (mapbox.com). Las imágenes de la aplicación con mas detalles cartográficos se encuentran disponibles en el repositorio de Vector Point:** <https://github.com/chirimacha/VectorPoint>.

**Mapas de riesgo de infestación**

La función principal de VectorPoint es el mapa de riesgo (Figura 1), el cual muestra los estimados del riesgo relativo de *T. infestans* generados por un modelo estadístico (descrito debajo) a un nivel de hogares individuales. El mapa tiene el propósito de ser usado por inspectores de salud quienes llevan a cabo la vigilancia de casa a casa. El resultado del modelo es presentado en un formato simple e intuitivo en el que los estimados de riesgo son divididos entre cinco cuantiles, extendiéndose de riesgo más bajo a más alto, y cada cuantil es representado en el mapa por un color. Utilizamos el esquema de cinco clases de color de varios tonos, ‘YlOrRd,’ desarrollado porBrewer [[74]](https://paperpile.com/c/0nGvWM/w3Sku) para la visualización de datos de cartografía [[75,76]](https://paperpile.com/c/0nGvWM/pTpX9+L8dZm), el cual es amigable para daltónicos. El esquema de color progresa de un amarillo claro a un rojo oscuro (una progresión asociada con peligro aumentado entre los hablantes de español [[77]](https://paperpile.com/c/0nGvWM/59roY)) con una saturación que aumenta con el riesgo de infección, y naranja representando un riesgo intermedio. Cada hogar aparece en el mapa como un punto que es coloreado con uno de los cinco colores que corresponde con su estimación de riesgo de infestación. Una leyenda en la esquina del mapa presenta los colores en compañía de su descripción en español de acuerdo con su categoría de riesgo de infestación correspondiente. Cada categoría de riesgo es presentada en el mapa de igual manera. Los mapas son diseñados para mostrar los riesgos relativos (i.e., el riesgo de infestación de un hogar relativo al de otros hogares en el barrio) pero pueden ser ajustados por los diseñadores para mostrar los datos divididos entre cualquier número de cuantiles, o para mostrar estimados de riesgo absolutos o relativos.

**Herramienta de Colección de Datos**

La segunda función de VectorPoint es ser práctica para colección de datos (Figura 2). Los inspectores pueden añadir información de los resultados de una inspección individual del hogar directamente en un formulario de la aplicación. Este formulario está diseñado para colectar la misma información utilizada por los formularios físicos del Ministerio de Salud en la vigilancia de *T. infestans:* fecha, código del hogar (explicado a continuación), áreas del hogar que fueron inspeccionadas (intradomicilio, peridomicilio, ambos), número de habitantes, número y tipo de animal doméstico, y si habían o no rastros de *T. infestans* (generalmente, huevos, heces o exhuvias). Botones y menús desplegables son proveídos en lo posible para aumentar la consistencia, y para evitar errores tipográficos. Luego de la conclusión de este formulario, la información es encriptada y transmitida a una base de datos en SQL, eliminando el paso de la digitación de la información en forma física.

**Figura 2. Parte del formulario en VectorPoint para colección de datos.**

Debemos notar que la información de la vigilancia de *T. infestans* es organizada con códigos de identificación de cuatro números, asignados a cada hogar por el Ministerio de Salud Peruano al comienzo de la campaña de control de vectores. Los cuatro números consisten en códigos para: provincia/distrito/localidad/hogar. (VectorPoint es diseñado para el uso en vigilancia de casa a casa al nivel de la localidad, o barrios de 30 a 2000 hogares.) Hemos mantenido el sistema de ID de cuatro números en VectorPoint, y a través del manuscrito nos referimos a ese como códigos de hogares.

## **Modelo espacio-temporal**

El modelo en VectorPoint está diseñado para estimar la probabilidad relativa de la infestación de *T. infestans* en hogares en un paisaje urbano. El modelo incorpora tres tipos de información: (i) covariantes del sitio, (ii) los resultados de inspecciones previas por *T. infestans*, y (iii) la historia de infestación en sitios vecinos. Para cada sitio, incluimos un covariante que es un indicador de la participación del hogar en la fase de ataque de la campaña de control, durante la cual el insecticida fue aplicado a todos los hogares participantes, ya que estudios previos demuestran que los hogares que no participaron son más probables de ser infestados [[30]](https://paperpile.com/c/0nGvWM/TFDeU). No incluimos otros factores a escalas más detalladas para los riesgos de infestaciones de *T. infestans,* tales como crianza de cuyes [[23]](https://paperpile.com/c/0nGvWM/d3jOm), debido a que la información no estaba disponible a la escala requerida para la aplicación.

Concretamente, dada la probabilidad de presencia de vectores, *i,* al tiempo, *t*, debe ser dada por$\pi_{i}$. Diseñamos el modelo de probabilidad usando un modelo logístico con intercepto, $\beta_{0}$, información de covariantes${, \beta}_{1}$, y efectos aleatorios separables de tiempo y lugar$,u_{it} y v_{it}:$

$$logit(\pi_{i})= \beta_{0}+ \beta_{1}+ u_{it}+ v_{it}$$

que $u_{it}$es la realización del campo Gaussiano con una estructura de covariación Matérn [[78,79]](https://paperpile.com/c/0nGvWM/aD3Mk+VYV5s). El campo Gaussiano funciona de modo que cualquier ajustamiento a la estimación para un hogar afecta todos los hogares en un área específica, con un efecto mayor en aquellos cercanos. La función Matérn es un modelo versátil de covariancia que incluye covariancia Gaussiana como un caso especial [[80]](https://paperpile.com/c/0nGvWM/6TSOw). El término$v_{it}$ es un efecto aleatorio autoregresivo discreto de primer orden.

Como mencionado previamente, el modelo toma en cuenta la historia de inspección para cada hogar/sitio. Actualmente incluimos cuatro periodos discretos (Figura 3). Seleccionamos nuestros periodos en referencia con la fase de control de vectores en la campaña en cada área. El periodo más temprano refleja la fase de ataque de la campaña, la cual ocurrió entre enero 7, 1997 y enero 6, 2014, dependiendo del distrito. El segundo periodo es el de vigilancia temprana, e incluye todos los datos recolectados entre enero 7, 2014 y enero 6, 2016. El tercer periodo incluye información de inspecciones realizadas entre enero 7, 2016 y enero 4, 2018. El último periodo refleja el actual año, de enero 5, 2018 al presente. Los últimos periodos pueden ser ajustados si es necesario. La probabilidad de infestación prevista para el tiempo más reciente es visualizada en la aplicación.

**Figura 3. Mapa de la localidad tal como está representada en el modelo a través de los cuatro periodos(T1-T4). Los puntos representan los hogares. T1 representa los periodos de tiempo de la fase de ataque de la campaña de control, la cual ocurrió entre enero 11, 1997 y enero 6, 2014, dependiendo del distrito. En T1, los puntos grises representan hogares que participaron en la fase de ataque de la campaña de control, pero no tenían evidencia de infestación de *Triatoma infestans,* y los puntos negros representan hogares que participaron y estaban infestados. Los periodos de tiempo T2-T4 representan el periodo de tiempo de la fase de vigilancia (T2: Enero 7, 2014 - Enero 6, 2016, T3: Enero 7,2016 - Enero 4,2018, T4: el año actual, actualmente definido por Enero 5,2018 - presente). En T2-4, los puntos grises representan los hogares que fueron inspeccionados y no estaban infestados con *T. infestans* durante la fase de vigilancia.**

Realizamos este modelo con aproximaciones integradas de Laplace, usando el paquete de R, “INLA”[[81,82]](https://paperpile.com/c/0nGvWM/j9FN4+SPwCk). Para considerar los efectos de las calles como barreras semi-permeables para la expansión de *T. infestans* [[29]](https://paperpile.com/c/0nGvWM/3vSSI), utilizamos una extensión de un modelo de campo Gaussiano en el cual extendimos el mapa de la ciudad de manera que su centro geográfico (i.e., el promedio estadístico de las coordenadas) de cada bloque de la ciudad se encuentra a un múltiplo (1.5;[[83]](https://paperpile.com/c/0nGvWM/56VUa)) de su distancia verdadera. Mantenemos la estructura de cada bloque, de manera que sólo la distancia entre bloques es extendida [[83]](https://paperpile.com/c/0nGvWM/56VUa). Definimos priores determinados (la media = 1.17 y la desviación estándar = 0.01) en el covariante de no haber participado en la campaña original de aplicación de insecticida, basados en nuestro análisis previo de este factor [[30]](https://paperpile.com/c/0nGvWM/TFDeU). Definimos priores en el término intercepto para corresponder a una prevalencia de infestación base de aproximadamente uno en 1000, con la matriz de precisión de 50. Este valor refleja nuestra mejor estimación de la prevalencia de infestación de *T. infestans* en Arequipa basada en los resultados recientes de vigilancia pasiva (i.e., reportes de infestación de *T. infestans* por miembros de la comunidad que son luego confirmados por el personal de salud) y vigilancia activa (la vigilancia conducida por nuestro equipo y el Ministerio de Salud de casa a casa).

**Flujo de datos y plataforma**

Las estimaciones de riesgo de infestación generadas por el modelo son enviadas a una base de datos basada en la nube (Servicio de Bases de Datos Relacionales de Amazon de los Servicios Web Amazon) mediante el paquete RMySQL [[84]](https://paperpile.com/c/0nGvWM/VU2JQ). Esta información es enviada al servidor Shiny [[85]](https://paperpile.com/c/0nGvWM/Se3dv), el cual presenta los estimados de riesgo en la aplicación. Inversamente, los datos colectados en la aplicación son enviados a la base de datos SQL, e incorporados en la siguiente actualización del modelo. Presentamos un diagrama del flujo de datos de VectorPoint en la Figura 4.

**Figura 4. Diagrama de flujo de datos de VectorPoint. Desde la izquierda, las estimaciones de riesgo generadas por el modelo son enviadas primero a una base de datos en SQL, luego enviadas a la plataforma Shiny y el servidor para obtener la visualización en el mapa de riesgo. Desde la derecha, la información colectada por la aplicación es enviada al servidor Shiny y luego a la base de datos en SQL. El modelo luego obtiene la nueva información de la base de datos en SQL la próxima vez que sea actualizada. Todos los datos son encriptados en TLS.**

Para prever problemas de conexión, VectorPont tiene una función que guarda los resultados de inspección parciales, y las envía a los servidores cuando la conexión es restaurada. VectorPoint apoya múltiples tablas de resultados para modelos, y todas las operaciones son computadas en paralelo, lo cual le permite a la aplicación ser usada por múltiples inspectores simultáneamente sin la degradación a su velocidad.

Construimos VectorPoint utilizando software de acceso abierto para permitir su distribución y acceso exenciones. Lo construimos usando el paquete Shiny en R [[85]](https://paperpile.com/c/0nGvWM/Se3dv), y lo implementamos completamente en el paquete de acceso abierto del lenguaje de programación R [[82]](https://paperpile.com/c/0nGvWM/SPwCk). Visualizamos las predicciones de infestación de vectores en mapas utilizando el paquete Leaflet en R [[86]](https://paperpile.com/c/0nGvWM/0iZei) y luego superpusimos esta información sobre la información de las calles proveniente de OpenStreetMap.org. El código de acceso abierto y las herramientas relacionadas para VectorPoint pueden ser descargadas en: <https://github.com/chirimacha/VectorPoint>*,*en donde también se encuentra un link para la instalación completa y funcional de la aplicación, disponible en la sección: README.

**Flujo del trabajo en el campo**

VectorPoint se encuentra en un servidor web al cual se tiene acceso por medio de un navegador de internet en cualquier computador o dispositivo móvil, sin importar su plataforma (Android, iOS, Windows, OSX, Linux, etc.). Al llegar a la página de internet de VectorPoint, el inspector es presentado con unos pasos de autenticación en la que escriben un usuario y contraseña. Todas las conexiones son encriptadas, y a los mapas de riesgo sólo tiene acceso el equipo de estudio y el personal de salud autorizado. Luego de la autenticación del usuario, el inspector selecciona la localidad o grupo de localidades en las que llevarán a cabo su vigilancia del día. La aplicación les provee esta información y la adquiere de la base de datos y actualiza el mapa de riesgo correspondiente a estas localidades, con zoom alejado (Figura 1a). Esta vista provee al inspector una vista de alto nivel de los hogares de la localidad y sus riesgos relativos de infestación.

De aquí, el inspector puede acercar su vista en el mapa (Figura 1b) y selecciona un hogar para potencialmente visitarlo al hacer click en su punto correspondiente en el mapa. Una ventana de diálogo se abrirá incluyendo el código del hogar, la fecha en la que este hogar fue visitado por última vez, y si la casa fue inspeccionada en esta instancia. Si el inspector decide visitar este hogar, ellos pueden cargar un formulario de entrada de datos con el código del hogar y la fecha ya completadas. Si el inspector recibe permiso para inspeccionar este hogar por la presencia de *T. infestans,* la información de esta inspección es entrada en este formulario. Si el inspector no recibe permiso de inspeccionar el hogar, esto es registrado en el formulario como una de cuatro alternativas de resultados: “entrevista”, “cerrado”, renuente,” o “volver.” “Entrevista” significa que el inspector habló con alguien en la puerta del hogar acerca de la infestación de *T. infestan* pero no recibió permiso de entrar al hogar e inspeccionarla; “cerrado” se refiere a las instancias en que nadie respondió a la puerta; “renuente” significa que la inspección fue directamente rechazada por el habitante del hogar; y “volver” significa que quien se encontraba en el hogar le pidió al inspector que volviera luego. Luego de cada visita a un hogar, los datos son enviados desde la aplicación a la base de datos, independientemente del resultado de la visita. En los casos de apagones de datos o problemas de conexión al internet, la información de la inspección puede ser guardada en la aplicación y enviada a la base de datos luego, como es descrita anteriormente. Este proceso es repetido para cada hogar que es visitado por el inspector en un día determinado. Al final del día, todos los datos colectados con la aplicación son extraídos de la base de datos e incluidos en el modelo para generar nuevas predicciones. Estas predicciones son luego mandadas a la base de datos y visualizadas en el mapa.

## **Estudio de campo**

En el estudio que compara la vigilancia de *T. infestans*  con la aplicación con las prácticas actuales usando mapas dibujados a mano (Figura 5), ocho miembros de nuestro equipo de campo, quienes fueron entrenados para llevar a cabo las inspecciones de *T. infestans,* llevaron a cabo la vigilancia de vectores en Arequipa por un total de dos semanas de trabajo (10 días). Al principio de cada semana, los inspectores fueron asignados aleatoriamente a (i) a una zona para visitar y (ii) si utilizarían la aplicación o los mapas físicos en esta zona. Solo un inspector fue asignado a cada zona de búsqueda, las cuales se encuentran en el mismo distrito. Cada zona de búsqueda cumplió con los siguientes requisitos: (i) estaba localizada en un área desarrollada (i.e., las calles son pavimentadas) en la porción central del distrito, (la cual es más segura que las áreas periféricas y menos desarrolladas); (ii) contenía un mínimo de 400 hogares y no más de 1.25 veces el número de hogares en la zona con el número más bajo de hogares.; (iii) el área era de un mínimo de f 0.1 km^2^, y no podía ser más grande que dos veces el tamaño de la zona con el área menor; (iv) la densidad de los hogares debía ser por lo menos 20000 hogares por km^2^; y (v) la zona de búsqueda era en una localidad en donde por lo menos un hogar había tenido resultados positivos por *T. infestans* durante la fase de ataque de la campaña de control. Estos criterios resultaron en 16 zonas de búsqueda con 416-514 hogares, un área de 0.120.20 km^2^, un perímetro de 1.62-2.37 km, y una densidad de hogares en el rango de 2,570 a 3,623 hogares por km^2^. La mitad de los inspectores utilizaron la aplicación durante la primera semana y mapas físicos en la segunda semana, y la otra mitad utilizó los mapas físicos en la primera semana, y cambiaron al uso de la aplicación en la segunda semana. Todos los inspectores utilizaron el mismo modelo de teléfono y sistema operativo cuando usaron la aplicación (Samsung Galaxy 7, con la versión Android 7.0) para controlar variaciones entre dispositivos. Los inspectores recibieron entrenamiento en el uso de la aplicación antes de comenzar este experimento.

**Figura 5. Ejemplo de un mapa físico que se usa en la vigilancia de vectores como en la práctica actual. Los números en cada bloque son los últimos tres dígitos del código del hogar, con flechas indicando el orden ascendente. Por ejemplo, un bloque con ‘1 → 12’ indica que los hogares con códigos uno a 12 se encuentran en este bloque. Los nombres de las calles y otra información para identificación han sido removidos.**

La vigilancia fue llevada a cabo de manera diaria durante los días de trabajo regulares (lunes a viernes) y horas (7am-1pm). Los inspectores fueron instruidos a llevar a cabo la vigilancia de *T. infestans* tal como lo hacían previamente y que su objetivo era simplemente encontrar *T. infestans,* para evitar las percepciones de que tenían que cumplir con una cuota de visitar a cierto número de casas. El protocolo para las inspecciones de *T. infestans* es estipulado por el Ministerio de Salud Peruano, y consiste en búsquedas sistemáticas en todas las áreas de domicilio y peridomicilio (pendiente de el permiso de los residentes), incluyendo espacios ocupados por humanos y animales. Estas búsquedas duran aproximadamente una hora; se permite flexibilidad para tener en cuenta la heterogeneidad en el tamaño de los hogares. Durante la búsqueda, el inspector busca *T. infestans* vivos, huevos, heces y exhuvias de *T. infestans*.

Los inspectores que usaron los mapas de papel algunas veces tuvieron acceso a dos piezas de información de riesgo, ya que cada código de hogar (pintado afuera de la casa) contiene indicadores de participación en la fase de ataque de la campaña de control de vectores, y un signo ´+´al final del código si en alguna instancia se encontraron infestaciones de *T. infestans* en el hogar. Esta información sólo puede ser vista cuando los inspectores se paran al frente de los hogares mirando el código del hogar; no está presente en los mapas físicos, lo cual indica que los inspectores que usaron los mapas físicos no podían ver una distribución espacial de los hogares con estos factores de riesgo. Adicionalmente, muchos de los códigos de los hogares han sido pintados sobre la versión original por los dueños de esos hogares al mejorar sus viviendas después de la fase de ataque de la campaña, lo cual resulta en la incapacidad de identificar cada uno de los hogares con estos indicadores de riesgo simplemente al observar este código.

**Análisis de datos**

Para medir el efecto del uso de la aplicación de campo en la productividad de los inspectores**,** comparamos el número total de hogares visitados entre los dos medios. Seleccionamos estas medidas para probar si la aplicación estaba disminuyendo en velocidad o restringiendo sus actividades de vigilancia debido a dificultades técnicas u otras. Como mencionado anteriormente, no comparamos el número de hogares infestados en estas visitas, ya que la prevalencia de infestación es actualmente muy baja [[29]](https://paperpile.com/c/0nGvWM/3vSSI).

Para investigar si los inspectores utilizaron la información de riesgo proveída por la aplicación en su selección de hogares para visitar, comparamos la proporción de hogares visitados que eran hogares de alto riesgo (los dos niveles más altos de riesgo) cuando usaron la aplicación y con el uso de mapas físicos. Para investigar el uso de esta información aún más, comparamos la proporción total de hogares visitados que fueron inspeccionados entre la aplicación y los mapas de papel entre aquellos hogares que de alto riesgo que fueron visitados (los hogares presentados en la aplicación como ´riesgo más alto´, y ´alto riesgo´) y sólo los hogares de bajo riesgo (hogares presentados en la aplicación como ´intermedio,´ ´riesgo bajo,´ y ´riesgo más bajo´). Estábamos interesados en el efecto de la posesión de información acerca del riesgo estimado de infestación para un hogar y su potencial influencia en el resultado de la visita (i.e., si el hogar fue inspeccionado, cerrado, la inspección fue rechazada, una entrevista fue llevada a cabo, o si el residente pidió que el inspector volviera luego).

Finalmente, averiguamos por evidencia cualitativa acerca del uso de la información de riesgo por medio de la comparación de los patrones de desplazamiento de los inspectores a través de las zonas de búsqueda cuando usaban la aplicación y cuando usaban los mapas de papel. Los mapas diarios para cada inspector están localizados en el repositorio de Vector Point, (<https://github.com/chirimacha/VectorPoint>). Examinamos los patrones de desplazamiento a menor escala, tales como los cambios en dirección y tendencia en visitar un hogar vecino versus el intercalar la visita a hogares. Adicionalmente miramos a los patrones a gran escala, tales como las tendencias a visitar todos los hogares de una zona versus visitar varios hogares a través de diferentes áreas, al igual que el desplazamiento acumulativo a través del área total de la zona de búsqueda a lo largo de los cinco días, el cual es referido como ´cubrimiento espacial´ de la zona.

**Pruebas estadísticas**

Para comparar el número total de hogares visitados y los hogares inspeccionados entre la aplicación y los mapas físicos usamos una prueba de tipo t. Para los análisis que envolvían el nivel de riesgo del hogar, dividimos los hogares visitados entre categorías binarias de riesgos más altos o más bajos, consistiendo en dos niveles de más alto riesgo y estos de los tres niveles más bajos, respectivamente. Para los análisis que envolvían el resultado de la visita, clasificamos los resultados de la visita entre categorías binarias ´inspección´, y ´otro.´ Para todas las medidas excepto el total de hogares visitados y el total de hogares inspeccionados, llevamos a cabo un análisis preliminar usando la prueba exacta de Fisher para medir las diferencias entre la aplicación y los mapas de papel para cada inspector, y una prueba de Chi-cuadrada de Mantel-Haenzel con corrección de continuidad para examinar el cambio global unidireccional entre todos los inspectores. Llevamos a cabo un análisis secundario usando Modelos Mixtos Lineales y Generalizados con “ID de Inspector” interceptos aleatorios [random intercepts] para probar si el efecto fijo de la inclusión de una aplicación ayudaba a explicar cualquier varianza. En este análisis comparamos los resultados de un modelo con interceptos aleatorios de ID de inspector con los resultados de un modelo con estos interceptos aleatorios de ID de Inspectores y una adición del efecto fijo de la aplicación. Usamos los puntajes BIC [[87]](https://paperpile.com/c/0nGvWM/XTSG) como medida para evaluar si la adición del efecto fijo de la aplicación mejoró el rendimiento de los modelos, con valores de BIC más bajos que sugerían mejoras en el modelo. Adicionalmente, evaluamos las cuotas de probabilidades (‘odds ratios’ u ‘OR’) en los modelos de efectos fijos de la aplicación para entender si la aplicación aumentó los chances de que el resultado fuera evaluado. Los modelos fueron ajustados por la probabilidad máxima (Aproximación Laplace) usando la función ´glmr´ en el paquete lme4 [[88]](https://paperpile.com/c/0nGvWM/Oh3eu) para R. Todos los análisis de datos fueron llevados a cabo en el medio de computación estadística de R [[82]](https://paperpile.com/c/0nGvWM/SPwCk).

**Resultados**

En el curso de diez días, ocho inspectores visitaron un total de 2,119 hogares, de los cuales**,** 767 fueron inspeccionados por *T. infestans* (Tabla 1). En los cinco días en los que se usaron mapas de papel, 1,081 hogares fueron visitados, los cuales resultaron en 366 inspecciones (33.9%). En los cinco días del uso de la aplicación 1,038 hogares fueron visitados, resultando en 401 inspecciones (38.6%).

| **Inspector** | **Total de Visitas** | | **Inspecciones** | |
| --- | --- | --- | --- | --- |
|  | ***Aplicación*** | ***Mapas físicos*** | ***Aplicación*** | ***Mapas físicos*** |
| A | 88 | 90 | 44 | 37 |
| B | 184 | 141 | 65 | 49 |
| C | 164 | 175 | 31 | 19 |
| D | 111 | 95 | 81 | 76 |
| E | 130 | 123 | 46 | 43 |
| F | 126 | 145 | 47 | 43 |
| G | 112 | 114 | 46 | 56 |
| H | 123 | 198 | 41 | 43 |
| Total | 1,038 | 1,081 | 401 | 366 |
| **Tabla 1. Columnas: número total de casas visitadas (izquierda) y número de casas visitadas e inspeccionadas (derecha) con el uso de la aplicación y mapas físicos. Filas: datos para cada inspector.** | | | | |

**Efecto de la aplicación en la productividad de los inspectores**

No encontramos una diferencia en el número total de hogares visitados entre el uso de la aplicación y los mapas físicos (prueba t, p = 0.67, Tabla 1), el número de hogares que fueron inspeccionados (prueba t, p = 0.17, Table 1 ), o la proporción de visitas que resultaron en inspecciones (prueba Mantel-Haenszel, chi cuadrado = 2.63, p = 0.105, odds ratios (OR) = 1.18, 95% CI = 0.97- 1.42, Figura 6), lo cual sugiere que la aplicación no redujo la productividad en el campo. Al estudiar los números específicos para los inspectores, ninguno de ellos inspeccionó más hogares con el uso de mapas físicos. Dos inspectores, C y H, inspeccionaron una proporción significativamente más alta cuando usaron la aplicación basados en los valores p (prueba exacta de Fisher, Inspector C: p = 0.046, OR= 1.91, 95% CI = 0.99-3.76; Inspector H: p = 0.026, OR = 1.80, 95% CI =1.05-3.08, Figura 6).

En los modelos mixtos lineales generalizados, la cuota de probabilidades del efecto fijo (‘odds ratio’) de la aplicación fue 1.18 (95% CI = 0.97-1.42, Tabla 2), lo que sugiere el poco efecto de la aplicación en la probabilidad de que un hogar visitado fuera inspeccionado. La desviación estándar en los efectos aleatorios de inspectores fue alta**,** independientemente de la inclusión del efecto de la aplicación en el modelo (SD = 0.76, 95% CI = 0.49-1.39 con la aplicación; SD = 0.77, 95% CI = 0.49-1.40 sin la aplicación). El uso del efecto fijo de la aplicación no mejoró el modelo, ya que ambos modelos tuvieron puntajes de BIC similares (138.71 y 138.80 para el modelo con y sin la aplicación, respectivamente). Los resultados de los modelos mixtos lineales generalizados son presentados en la Tabla 2.

**Figura 6. Distribuciones de los resultados de visitas. Las inspecciones se encuentran en azul, y otros resultados son mostrados en gris. *La proporción de hogares significativamente más alta cuando se usó la aplicación (prueba exacta de Fisher, p<0.05). La leyenda se traduce como: “Resultado de Visita; Inspección; Cerrada; Renuente; Volver; Entrevista.”**

**Uso de la información de riesgo**

Cinco de los ocho inspectores (B,D,E,F, y H; 62.5%, Figura 7), visitaron más hogares de alto riesgo (los dos niveles más altos) cuando usaron la aplicación que cuando usaron mapas físicos (prueba exacta de Fisher, Inspector B: p = 4.14e-14, OR = 25.38, 95% CI = 7.80-131.06; Inspector D: p = 1.81e-14, OR=40.71, 95% CI = 9.94-360.00; Inspector E: p = 4.82e-07, OR = 4.42, 95% CI = 2.37-8.51; Inspector F: p < 2.2e-16, OR = 10.10, 95% CI = 5.58-18.80; Inspector H: p < 2.2e-16, OR = 14.86, 95% CI = 6.80-37.13). Dos inspectores (25%), A and C, no tuvieron diferencias en la proporción de visitas totales que eran de alto riesgo cuando usaron la aplicación (prueba exacta de Fisher, Inspector A: p = 0.65, OR = 1.19, 95% CI = 0.63-2.25; Inspector C: p = 0.12, OR = 1.43, 95% CI = 0.90-2.26; Figure 7), y uno de los inspectores (G), visitó más hogares de alto riesgo cuando utilizó mapas de papel (prueba exacta de Fisher, p < 2.2e-16, OR = 0.0, 95% CI = 0.0-0.02; Figura 7). En general, hubo un cambio positivo en el nivel de riesgo de los hogares visitados con el uso de mapas físicos al uso de la aplicación (prueba Mantel-Haenszel, chi cuadrado = 104.44, p < 2.2e-16, OR = 2.42, 95% CI = 2.00-2.92; Figura 7) lo que sugiere que los inspectores incorporaron la información de riesgo provista por la aplicación en su selección de hogares a visitar.

En el modelo mixto lineal generalizado, el efecto de la aplicación fue un predictor significativo de un hogar de alto riesgo que sería visitado (p < 2e-16), y aumentó la probabilidad de visitar un hogar de alto riesgo por 2.73 (95% CI = 2.24-3.32, Tabla 2). La desviación estándar en los efectos aleatorios de ID de inspectores fue alta en los dos modelos (0.76, 95% CI = 0.48-1.37 con la aplicación, y 0.77, 95% CI = 0.50-1.20 sin la aplicación). Los puntajes de BIC sugieren que la aplicación mejoró el modelo, ya que fueron 442.9 y 544.3 para el modelo con y sin la aplicación, respectivamente.

|  | **Productividad de los inspectores** | | **Uso de información de riesgo** | |
| --- | --- | --- | --- | --- |
|  | ***Variación aleatoria de inspector*** | ***Con el efecto fijo de aplicación*** | ***Variación aleatoria de inspector*** | ***Con el efecto fijo de aplicación*** |
| **Variable dependiente** | # inspecciones | | # hogares de alto riesgo visitados | |
| **Variable predictiva** | Variación de inspectores | Uso de la aplicación | Variación de inspectores | Uso de la aplicación |
| **Intercepto de variación de inspectores**  **(95% CI)** | -0.495  (-1.107-0.118) | -0.576  (-1.191-0.041) | 0.571  (-0.031-1.177) | 0.110  ( -0.512-0.733) |
| **SE del Intercepto** | 0.275 | 0.279 | 0.272 | 0.281 |
| **Valor p de intercepto** | 0.0723 | 0.0387 | 0.0356 | 0.697 |
| **Estimación de parámetro del efecto de la aplicación**  **(95% CI)** | n/a | 0.163  (-0.026-0.352) | n/a | 1.00  (0.808-1.202) |
| **SE de la estimación de efecto de la** **aplicación** | n/a | 0.096 | n/a | 0.100 |
| **Valor p de efecto de la aplicación** | n/a | 0.0906 | n/a | <2e-16 |
| **Probabilidad promedio estimada para la ocurrencia del evento**  **(perfil de probabilidad 95% CI)** | 0.379  (0.249-0.530) | 0.398  (0.228- 0.597) | 0.639  (0.492-0.765) | 0.753  (0.573-0.874) |
| **SD de la estimación de efectos aleatorios**  **(95% CI)** | 0.767  (0.489-1.395) | 0.764  (0.487- 1.391) | 0.756  (0.484-1.374) | 0.774  (0.495-1.202) |
| **Odds ratio**  **(95% CI)** | n/a | 1.18  (0.974-1.142) | n/a | 2.73  (2.240-3.321) |
| **Puntaje BIC** | 138.80 | 138.71 | 544.30 | 442.90 |
| **¿Modelo mejorado con el efecto de la aplicación?** | no | | si | |
| **Tabla 2. Resultados de los modelos mixtos lineales generalizados investigando la productividad de los inspectores y el uso de la información de riesgo con y sin la aplicación VectorPoint como efecto fijo.** | | | | |

No observamos ningún patrón asociado con el orden de brazos (i.e., cuál medio fue utilizado en la primera semana y la segunda semana, Figura 7). De los cinco inspectores que visitaron una proporción más alta de hogares de alto riesgo usando la aplicación, dos usaron la aplicación primero, y tres usaron los mapas físicos primero. En el caso de los dos inspectores que no mostraron diferencias entre la aplicación y los mapas, uno empezó con la aplicación y el otro con el mapa físico. El inspector que visitó más hogares de alto riesgo con el mapa físico usó la aplicación primero.

Tampoco encontramos diferencias entre la aplicación y el mapa físico en la proporción de visitas a hogares de alto o bajo riesgo que resultaron en inspecciones, lo que sugiere que no hay una asociación entre el conocimiento del riesgo de infestación de un hogar y el resultado de permiso para una inspección (prueba exacta de Fisher, p > 0.05 para todos los inspectores en los hogares de alto y bajo riesgo; en general: prueba de Mantel-Haenszel, hogares de alto riesgo: Chi cuadrado1.71, p = 0.19, OR común = 1.19, 95% CI = 0.93-1.51; hogares de bajo riesgo: Chi cuadrado= 0.44, p = 0.51, OR común= 1.18, 95% CI = 0.78-1.78).

**Figura 7. Distribución de riesgo de infestación de todos los hogares visitados por cada inspector. Los superíndices en el eje x indican el orden del brazo (cuál medio se usó en la primera semana y cuál medio se usó en la segunda semana); los valores < 4% no están marcados debido a límites espaciales; *p < 0.001, con más hogares de alto riesgo visitados con la aplicación; ^p < 0.001, con más hogares de alto riesgo visitados con los mapas físicos.**

## **Caracterización cualitativa de patrones de desplazamiento y cobertura espacial**

## Observamos dos patrones de desplazamiento predominantes a través del estudio. El primero se enfocó en los hogares individuales, mientras que el segundo se enfocó en la cobertura de la zona de búsqueda. (Tal como mencionado anteriormente, los mapas diarios de los inspectores se encuentran disponibles en el repositorio de VectorPoint [<https://github.com/chirimacha/VectorPoint>].) Mientras la mayoría de los inspectores exhibieron un patrón o el otro, algunos inspectores mostraron una mezcla de los dos durante la semana. En el patrón enfocado en hogares individuales, los inspectores tendieron a viajar distancias cortas (1-3 bloques /día), y visitaron todos los hogares en el bloque. Los patrones de desplazamiento parecían sistemáticos y enfocados en cubrir todos los hogares en un área pequeña de la zona, lo cual resultó en una baja cobertura espacial de la zona de búsqueda total. En el patrón que era más enfocado en la cobertura de la zona de búsqueda, los inspectores tendieron a viajar distancias más largas, visitando un grupo de casas en un área y luego desplazándose a otra sección de la zona. El desplazamiento parece menos sistemático que el del patrón de enfoque individual, y el cual estaba enfocado en la zona entera de búsqueda, y el que resultó en una cobertura espacial más alta. Existen varios límites prácticos en el campo de estudio que influencian los desplazamientos de los inspectores, pero los desplazamientos grandes a través de la zona fueron más comunes en inspectores que se enfocaron en la cobertura más amplia de la zona de búsqueda.

Siete de los ocho inspectores (A, B,C,D,E,F y H) demostraron por lo menos una indicación del uso de la información de riesgo en el contexto de estos patrones de desplazamiento predominantes. En el caso de los inspectores con patrones de desplazamiento enfocados en las casas individuales, la información de riesgo de infestación fue utilizada cuando saltaron hogares de bajo riesgo en medio de hogares de alto riesgo, o hicieron cambios abruptos en la dirección de su trayectoria para evadir un grupo de hogares de bajo riesgo, lo cual resultó en algunos casos en el aumento de la cobertura espacial de la zona de búsqueda. Mientras ellos usaban los mapas físicos, estos inspectores raramente saltaron hogares o cambiaron dirección, y su cubrimiento espacial de la zona fue bajo. Los inspectores que estaban más enfocados en la cobertura de la zona entera tendieron a indicar el uso de la información de riesgo con desplazamiento que era más sistemático y dirigido hacia los hogares de alto riesgo cuando usaron la aplicación. Cuando usaron la aplicación en áreas con grupos de hogares de alto riesgo, algunos inspectores ajustaron sus patrones de desplazamiento de amplio rango a unos más específicos y enfocados en los hogares individuales, lo que resultó en visitas a todos los hogares que se encontraban en un bloque de alto riesgo.

Observamos indicaciones de uso de información de riesgo en los inspectores que visitaron significativamente más hogares de alto riesgo con la aplicación (B,D,E,F,H) y en aquellos que no visitaron significativamente más hogares de alto riesgo con la aplicación (A and C). El Inspector C no visitó más hogares de alto riesgo, pero sí saltó grupos de hogares de bajo riesgo en algunos días, lo que sugiere que estaba ´probando ´la información de riesgo. El inspector A aumentó su cobertura espacial cuando usó la aplicación en comparación con su uso de mapas físicos, debido a un patrón de desplazamiento que parece estar ´probando´ los hogares de cada nivel de riesgo cada día. A lo largo de la semana el inspector procedió en orden ascendente de nivel de riesgo visitando hogares del riesgo más bajo en el primer día y finalizando en el día 5 con visitas a los hogares de más alto riesgo. Este inspector no visitó más hogares de alto riesgo usando la aplicación, pero parece que prestó atención a la información de riesgo de la aplicación. De hecho, al final de la semana, el inspector saltó los hogares de menor riesgo.

Solo un inspector (G) no presentó indicaciones del uso de la información de la aplicación en su selección de hogares a visitar. De hecho, este inspector visitó inesperadamente más hogares de alto riesgo cuando utilizó el mapa físico, lo cual atribuimos al agrupamiento de hogares del mismo nivel de riesgo en la zona de búsqueda. Basados en el examen de los patrones de desplazamiento del inspector, vimos que esto ocurrió debido a que el inspector demostraba tendencias fuertes hacia el desplazamiento de escala fina (enfocada en los hogares individuales) y seleccionó su punto de comienzo basado en el código del hogar (los códigos más bajos primero), y procediendo a través de la zona en orden numérico. Coincidentemente, el hogar en el que comenzó se encontraba al comienzo de un grupo grande de hogares de alto riesgo, lo que resultó en las visitas de solamente hogares de alto riesgo realizadas con el mapa físico.

**Discusión**

Presentamos una aplicación móvil diseñada para dar la oportunidad de incorporar información de riesgo basada en datos colectados en la vigilancia del vector *Triatoma infestans,* vector de la enfermedad de Chagas en la ciudad de Arequipa, Perú. En este estudio comparamos la vigilancia con el uso de la aplicación con la práctica actual en la que se usan mapas físicos. Observamos indicaciones múltiples de que la información de riesgo proveída en la aplicación fue utilizada en la selección de hogares para inspeccionar, lo que sugiere que la aplicación es una herramienta viable para mejorar la vigilancia del vector y el apoyo del proceso de toma de decisiones en el campo.

**mHealth de vanguardia**

Herramientas de mHealth, definidas como tecnologías móviles e inalámbricas para objetivos de salud [[89]](https://paperpile.com/c/0nGvWM/J0Ipg) han sido introducidos a un ritmo rápido [[90]](https://paperpile.com/c/0nGvWM/wTpCw). Mientras la mayoría de aplicaciones mHealth son para el uso personal relacionado con aspectos de salud individual, el uso de estas aplicaciones para la vigilancia de enfermedades en situaciones de recursos limitados ha crecido constantemente. El gran número de aplicaciones para la colección de datos disponibles para la vigilancia de enfermedades ha proveído la oportunidad de reemplazar sistemas complicados de colección de datos por medio de papel y lápiz, por medio de mecanismos que permiten que la información recolectada sea mandada a una base de datos central a la hora de la colección. Idealmente, el cambio de papel a tecnología llevará a un aumento de cobertura y exhaustividad en situaciones de recursos limitados, un paso crítico en el éxito de conseguir las metas de vigilancia [[91]](https://paperpile.com/c/0nGvWM/MCxLK). (Por supuesto, las nuevas tecnologías traen nuevos desafíos, algunos de los cuáles están descritos a continuación.) El paso siguiente es integrar la funcionalidad de colección de datos con las herramientas más complejas que soportan la toma de decisiones en el campo a tiempo real, o cerca del tiempo real. Mientras varias aplicaciones de colección de datos ofrecen capacidades

de visualización tales como mapas simples o gráficos de barras que muestran las distribuciones de datos brutos, estas funciones no están disponibles inmediatamente para los individuos que recolectan los datos en el campo, y su utilidad es limitada para el proceso de toma de decisiones. La aplicación VectorPoint es una de las primeras aplicaciones con funciones duales de modelos predictivos y visualización espacial de datos, los cuales tienen la intención de ayudar al usuario que recolecta los datos.

**Uso de la información de riesgo**

Como lo mencionado anteriormente, los inspectores con años de experiencia y rutinas establecidas tienen que adaptar su proceso de toma de decisiones incluyendo por lo menos alguna información acerca de riesgo proveída por la aplicación para que el objetivo de integrar los datos en las actividades de vigilancia sea cumplido. Las estimaciones generadas por el modelo no son más valiosas que el conocimiento derivado por la experiencia en el campo, y VectorPoint está diseñado para complementar la experiencia del inspector, no reemplazarla. En el estudio, observamos múltiples indicaciones de que los inspectores estaban utilizando la información de riesgo en la aplicación. Es decir, observamos un cambio positivo significativo en todos los inspectores en el número de hogares de alto riesgo que fueron visitados cuando se usó la aplicación, y la mayoría de inspectores visitaron más hogares de alto riesgo cuando utilizaron la aplicación. Estos resultados fueron confirmados en un análisis secundario comparando modelos mixtos lineales generalizados con y sin la aplicación, en los cuales la aplicación fue un predictor grandemente significativo del número mayor de hogares de alto riesgo visitados. En los mapas del desplazamiento de inspectores a través de las zonas de búsqueda, también observamos cambios en los patrones de desplazamiento predominantes en el uso de la aplicación. Aún los inspectores que no visitaron más hogares de alto riesgo con la aplicación mostraron evidencia de ‘probar’ la técnica de dirigir su enfoque hacia los hogares de alto riesgo o exploraron los diferentes niveles de riesgo, lo que sugiere que, con tiempo, los inspectores introducirán la información de riesgo gradualmente en sus actividades de vigilancia.

Mientras los resultados son alentadores, uno de los inspectores no mostró ninguna señal de uso de la información de riesgo, y no podemos asumir que la adopción de este modelo sería universal. De hecho, nuestros análisis más rigurosos que usan modelos mixtos lineales generalizados confirmaron que la variación entre inspectores es alta, independientemente de los resultados que son examinados. La disponibilidad para usar la información de la aplicación puede ser asociada con factores específicas a este individuo, tales como la edad o la experiencia con la tecnología [[89]](https://paperpile.com/c/0nGvWM/J0Ipg), y puede que tengamos que tener en cuenta la información demográfica de los inspectores al analizar el uso de la aplicación en estudios futuros para desarrollar estrategias para aumentar la adopción de esta técnica. Adicionalmente, la motivación para utilizar la aplicación puede aumentar a medida que más datos son recolectados, lo que nos permite comparar los índices de infestación entre hogares inspeccionados con la aplicación y con los mapas físicos. Tal como lo es mencionado previamente, el estudio no fue ajustado para comparar formalmente los índices de infestación; sólo una casa infestada fue encontrada en este estudio, y el inspector utilizó un mapa físico en esta instancia. En casos en los que inspectores tienen problemas en su empleo de la aplicación o si el entusiasmo por el uso de la nueva tecnología se desvanece con el tiempo, puede que el entrenamiento especializado o soluciones más creativas tales como incentivos [[92–94]](https://paperpile.com/c/0nGvWM/ewXAF+1CX5X+FN1Y5) o juegos [[95,96]](https://paperpile.com/c/0nGvWM/vXpAU+Ip1VP) sean necesarios.

**Cobertura espacial de las zonas de vigilancia**

A pesar de que la tendencia de viajar o no tendió a ser una característica fija en los dos medios del estudio, en algunos casos observamos cambios cualitativos en el cubrimiento espacial de la zona de búsqueda al usar la aplicación. Mientras la aplicación no es diseñada para responder al problema de cobertura espacial, se espera que el modelo redistribuya las estimaciones de riesgo relativo a través de la zona de búsqueda cada vez que es actualizado con nueva información. En otras palabras, si un hogar fue inspeccionado y no hubo rastros de infestación de *T. infestans,* el riesgo de infestación debería disminuir para todos los hogares a cierta distancia del hogar que no está infestado. De esta manera, los inspectores que utilizan la información de riesgo en la aplicación para dirigirse hacia los hogares de alto riesgo también mejorarán la cobertura de la zona. En este estudio, observamos este efecto sólo ocasionalmente, y en algunos casos, los inspectores que visitaron hogares de alto riesgo demostraron una cobertura disminuida en la zona de búsqueda cuando los hogares de alto riesgo estaban agrupados. Este resultado es probablemente debido a la cantidad pequeña de datos colectados por cada inspector y las cantidades tan variables de la información disponible en cada área de búsqueda antes del estudio. Tal como ha sido descrito anteriormente, esperamos que el agrupamiento de niveles de riesgo sea variable y dinámico, y que, en la ausencia de un hogar positivo, la distribución del riesgo se debería emparejar a medida que la información es colectada. La utilidad del modelo tras la aplicación, tal como todos los modelos Bayesianos, aumenta a medida que los datos son más acumulados.

Las extensiones para la aplicación o el modelo pueden ser útiles al mejorar la cobertura espacial de la vigilancia, y para explorar preguntas más complejas, tal como aquellas centradas en los beneficios o desventajas del uso de la aplicación. En particular, no sabemos si hay un intercambio entre exploración y explotación al usar esta aplicación; al enfocarnos en áreas que son conocidas o previstas para tener un riesgo alto de infestación (i.e., explotación), ¿fallaremos en detectar nuevos focos de alto riesgo (i.e., exploración)?

**Viabilidad: la superación de barreras logísticas y la adopción aumentada**

El uso de herramientas de mHealth en circunstancias de recursos limitados presenta desafíos inherentes [[50,55,73]](https://paperpile.com/c/0nGvWM/mhNJa+MjU32+Fyam7) debido a los requerimientos técnicos y financieros de los sistemas basados en dispositivos móviles. Una ventaja de VectorPoint es que utiliza tecnologías de acceso abierto, lo cual permite su uso a gran escala con costos bajos. Los otros costos operacionales incluyen la adquisición de los dispositivos móviles y sus suscripciones de datos celulares. Para aplicaciones que son basadas en la nube, tal Como VectorPoint, estas redes tienen que proveer un cubrimiento de datos de por lo menos velocidad 2G. En nuestros estudios de campo encontramos que los problemas de interrupción frecuente y la actualización de los mapas fueron evitados cuando la conexión era de velocidades de 3G o más altas. Afortunadamente, Perú presenta con una cultura móvil robusta, con 117 suscripciones celulares por cada 100 personas en 2016 [[97]](https://paperpile.com/c/0nGvWM/DmqoS), más altas que el promedio mundial de 101.5. Varias redes están disponibles a través del país, las cuales ofrecen planes de datos a altas velocidades. En los casos en los que problemas de conexión surgen, VectorPoint permite que los cambios en formularios y resultados sean guardados y actualizados cuando la red se restablezca, aunque cierta conexión de internet es necesaria para el uso eficiente de la aplicación. Actualmente, estamos trabajando hacia una versión completamente capaz de servir todas sus funciones sin necesidad de conexiones para superar esta limitación de VectorPoint. En nuestro estudio, no hubo diferencias en el número de casas visitadas entre el uso de mapas de papel y la aplicación, lo que sugiere que los problemas de conexión que sucedieron en el proceso no afectaron las actividades de vigilancia de manera negativa.

El ser una aplicación basada en la nube significa que VectorPoint puede funcionar en cualquier dispositivo sin ninguna instalación o configuración, pero requiere de la experiencia humana para vigilar y resolver sus componentes sistemáticos. Por suerte, actualmente se presenta una cultura creciente y la aceptación de herramientas de salud electrónicas en Perú, lo cual ha contribuido a la sostenibilidad de sistemas de información de salud en circunstancias de recursos limitados [[73]](https://paperpile.com/c/0nGvWM/mhNJa). Gozzer Infante [[98]](https://paperpile.com/c/0nGvWM/k0Obo) revisó 38 sistemas de salud que han sido introducidos en Perú entre 2002-2010, y 66% de estos todavía eran usados en 2015. Estos sistemas incluyen Alerta, un sistema de vigilancia de varias enfermedades [[99]](https://paperpile.com/c/0nGvWM/DFtfW); Netlab, un sistema de soporte laboratorio para el tratamiento contra el VIH [[100]](https://paperpile.com/c/0nGvWM/PeWbJ); y Magpi, una aplicación de colección de datos utilizada por investigadores para recolectar información acerca de la prevalencia de VPH [[61]](https://paperpile.com/c/0nGvWM/zDzOG). En el 2018, varias mHealth aplicaciones fueron confirmadas en su uso a nivel nacional (Leonardo Rojas, Instituto Nacional de Salud, comunicación personal) tales como Guardianes de la Salud [[101]](https://paperpile.com/c/0nGvWM/LR8uH), un sistema móvil de vigilancia a enfermedades para el uso en la visita Papal del 2018. En el caso de VectorPoint, colaboramos con personal local en el desarrollo de la aplicación desde su concepción, y es este personal local el que ahora controla su uso operacional y su ingeniería. Sin embargo, debemos mencionar que aunque VectorPoint es flexible, el modelo predictivo tiene que ser adaptado para dar cabido a las características epidemiológicas locales y ecológicas de cada escenario de vigilancia de enfermedades en el que es aplicado, lo que puede presentar desafíos ya que la experiencia y el conocimiento son limitados en algunos casos.

## **Conclusión**

Nuestros hallazgos sugieren que la aplicación VectorPoint podría ser útil para integrar evidencia y modelos en la vigilancia epidemiológica de enfermedades en ciudades. La aplicación fue diseñada para ser usada en la vigilancia e *T. infestans,* pero sus componentes son basados en la nube, de acceso abierto, y están listos para ser adaptados a otros escenarios apropiados, aunque la disponibilidad de suficiente información o recursos puede ser un obstáculo en algunos casos VectorPoint es fácil de usar, pero crítica en su función: ya que, sin estar vigilantes a los rastros y emergentes focos de vectores luego de una campaña de control de vectores, la transmisión de enfermedades retorna inevitablemente y puede revertir el progreso que se ha conseguido[[102]](https://paperpile.com/c/0nGvWM/MnSGo).

# **Reconocimientos**

Reconocemos con gratitud las contribuciones invaluables del Ministerio de Salud del Perú (MINSA), la Dirección General de Salud de las Personas (DGSP), la Estrategia Sanitaria Nacional de Prevención y Control de Enfermedades Metaxénicas y Otras Transmitidas por Vectores (ESNPCEMOTVS), la Dirección General de Salud Ambiental (DIGESA), el Gobierno Regional de Arequipa, la Gerencia Regional de Salud de Arequipa (GRSA), la Pan American Health Organization (PAHO/OPS), la Canadian International Development Agency (CIDA), yUptake Inc.

# **Referencias**

1. [Who. Chagas Disease Fact Sheet [Internet]. Geneva, Switzerland: World Health Organization; 2017. Available:](http://paperpile.com/b/0nGvWM/RgPTC) <http://www.who.int/mediacentre/factsheets/fs340/en/>

2. [Vos T, Barber RM, Bell B, Bertozzi-Villa A, Biryukov S, Bolliger I, et al. Global, regional, and national incidence, prevalence, and years lived with disability for 301 acute and chronic diseases and injuries in 188 countries, 1990-2013: A systematic analysis for the Global Burden of Disease Study 2013. Lancet. 2015;386: 743–800.](http://paperpile.com/b/0nGvWM/P21hI)

3. [World Health Organization. Chagas disease in Latin America: an epidemiological update based on the 2010 estimates. Weekly epidemiological record. 2015;6: 33–44.](http://paperpile.com/b/0nGvWM/7QbAT)

4. [Bern C. Chagas’ Disease. N Engl J Med. 2015;373: 456–466.](http://paperpile.com/b/0nGvWM/gVDEp)

5. [Rassi A Jr, Rassi A, Marin-Neto JA. Chagas disease. Lancet. Elsevier Ltd; 2010;375: 1388–1402.](http://paperpile.com/b/0nGvWM/jLUWW)

6. [Dias E. Estudos sobre o *Schizotrypanum cruzi*. Mem Inst Oswaldo Cruz. 1934;28: 1–110.](http://paperpile.com/b/0nGvWM/wqJZ7)

7. [Gorla D, Ponce C, Dujardin JP, Schofield CJ. Control strategies against Triatominae. In: Telleria J, Tibayrenc M, editors. American Trypanosomiasis Chagas Disease. 1st ed. Elsevier; 2010. pp. 233–245.](http://paperpile.com/b/0nGvWM/xicFn)

8. [Hashimoto K, Schofield CJ. Elimination of *Rhodnius prolixus* in Central America. Parasit Vectors. BioMed Central Ltd; 2012;5: 45.](http://paperpile.com/b/0nGvWM/AGAFu)

9. [Schofield CJ, Dias JCP. The Southern Cone Initiative against Chagas Disease. Adv Parasitol. 1999;42: 1–27.](http://paperpile.com/b/0nGvWM/4fhlN)

10. [Coura JR, Viñas PA. Chagas disease: a new worldwide challenge. Nature. 2010;465: S6–S7.](http://paperpile.com/b/0nGvWM/i42uD)

11. [Zeledon R. Chagas Disease: An Ecological Appraisal with Special Emphasis on its Insect Vectors. Annu Rev Entomol. 1981;26: 101–133.](http://paperpile.com/b/0nGvWM/jZRH3)

12. [Corredor Arjona A, Alvarez Moreno CA, Agudelo CA, Bueno M, López MC, Cáceres E, et al. Prevalence of *Trypanosoma cruzi* and *Leishmania chagas*i infection and risk factors in a Colombian indigenous population. Rev Inst Med Trop Sao Paulo. 1999;41: 229–234.](http://paperpile.com/b/0nGvWM/T2hEH)

13. [Feliciangeli MD, Sánchez-Martín MJ, Suárez B, Marrero R, Torrellas A, Bravo A, et al. Risk factors for *Trypanosoma cruzi* human infection in Barinas State, Venezuela. Am J Trop Med Hyg. 2007;76: 915–921.](http://paperpile.com/b/0nGvWM/wzUN1)

14. [Lardeux F, Depickère S, Aliaga C, Chavez T, Zambrana L. Experimental control of *Triatoma infestans* in poor rural villages of Bolivia through community participation. Trans R Soc Trop Med Hyg. 2014;109: 150–158.](http://paperpile.com/b/0nGvWM/DS4Va)

15. [Medina-Torres I, Vázquez-Chagoyán JC, Rodríguez-Vivas RI, de Oca-Jiménez RM. Risk factors associated with triatomines and its infection with *Trypanosoma cruzi* in rural communities from the southern region of the State of Mexico, Mexico. Am J Trop Med Hyg. 2010;82: 49–54.](http://paperpile.com/b/0nGvWM/EKenk)

16. [Bustamante DM, De Urioste-Stone SM, Juarez JG, Pennington PM. Ecological, social and biological risk factors for continued *Trypanosoma cruzi* transmission by *Triatoma dimidiata* in Guatemala. PLoS One. 2014;9. doi:](http://paperpile.com/b/0nGvWM/AKLkk)[10.1371/journal.pone.0104599](http://dx.doi.org/10.1371/journal.pone.0104599)

17. [Gurtler RE, Cohen JE, Cecere MC, Lauricella MA, Chuit R, Segura EL. Influence of humans and domestic animals on the household prevalence of *Trypanosoma cruzi* in *Triatoma infestans* populations in northwest Argentina. Am J Trop Med Hyg. 1998;58: 748–758.](http://paperpile.com/b/0nGvWM/A6JyQ)

18. [Coura JR. Chagas disease: what is known and what is needed A background article. Mem Inst Oswaldo Cruz. 2007;102: 113–122.](http://paperpile.com/b/0nGvWM/bGUs3)

19. [Ricardo-Silva AH, Lopes CM, Ramos LB, Marques WA, Mello CB, Duarte R, et al. Correlation between populations of Rhodnius and presence of palm trees as risk factors for the emergence of Chagas disease in Amazon region, Brazil. Acta Trop. 2012;123: 217–223.](http://paperpile.com/b/0nGvWM/K5seJ)

20. [Carrasco HJ, Segovia M, Londoño JC, Ortegoza J, Rodríguez M, Martínez CE. *Panstrongylus geniculatus* and four other species of triatomine bug involved in the *Trypanosoma cruzi* enzootic cycle: high risk factors for Chagas’ disease transmission in the Metropolitan District of Caracas, Venezuela. Parasit Vectors. 2014;7: 1003–1028.](http://paperpile.com/b/0nGvWM/N6Wm2)

21. [Gajate P, Pietrokovsky S, Abramo Orrego L, Pérez O, Monte A, Belmonte J, et al. *Triatoma infestans* in Greater Buenos Aires, Argentina. Mem Inst Oswaldo Cruz. 2001;96: 473–477.](http://paperpile.com/b/0nGvWM/FgM2T)

22. [Guzman-Tapia Y, Ramírez-Sierra MJ, Dumonteil E. Urban Infestation by *Triatoma dimidiata* in the City of Mérida, Yucatán, México. Vector-Borne and Zoonotic Diseases. 2007;7: 597–606.](http://paperpile.com/b/0nGvWM/ZSXhH)

23. [Levy MZ, Bowman NM, Kawai V, Waller LA, Geny J, Benzaquen EC, et al. Periurban *Trypanosoma cruzi*-infected *Triatoma infestans*, Arequipa , Peru. Emerg Infect Dis. 2006;12: 1345–1352.](http://paperpile.com/b/0nGvWM/d3jOm)

24. [Medrano-Mercado N, Ugarte-Fernandez R, Butrón V, Uber-Busek S, Guerra HL, De Araújo-Jorge TC, et al. Urban transmission of Chagas disease in Cochabamba, Bolivia. Mem Inst Oswaldo Cruz. 2008;103: 423–430.](http://paperpile.com/b/0nGvWM/UXSGo)

25. [Ricardo-Silva A, Monte-Gonçalves TC, Luitgards-Moura JF, Macedo-Lopes C, Pedrosa-da-Silva S, Queiroz-Bastos A, et al. *Triatoma maculata* colonises urban domicilies in Boa Vista, Roraima, Brazil. Mem Inst Oswaldo Cruz. 2016;111: 703–706.](http://paperpile.com/b/0nGvWM/1aaKU)

26. [Rodrigues VLCC, Pauliquevis Junior C, da Silva RA, Wanderley DMV, Guirardo MM, Rodas LAC, et al. Colonização de palmeiras por *Rhodnius neglectus* e invasão domiciliar em área urbana, Araçatuba, São Paulo, Brasil. Rev Inst Med Trop Sao Paulo. 2014;56: 213–218.](http://paperpile.com/b/0nGvWM/bltAg)

27. [Vallvé SL, Rojo H, Wisnivesky-Colli C. Urban ecology of *Triatoma infestans* in San Juan, Argentina. Mem Inst Oswaldo Cruz. 1996;91: 405–408.](http://paperpile.com/b/0nGvWM/9DVhs)

28. [Levy MZ, Barbu CM, Castillo-Neyra R, Quispe-Machaca VR, Ancca-Juarez J, Escalante-Mejia P, et al. Urbanization, land tenure security and vector-borne Chagas disease. Proceedings of the Royal Society B: Biological Sciences. 2014;281: 20141003–20141003.](http://paperpile.com/b/0nGvWM/4p641)

29. [Barbu CM, Hong A, Manne JM, Small DS, Quintanilla Calderón JE, Sethuraman K, et al. The Effects of City Streets on an Urban Disease Vector. PLoS Comput Biol. 2013;9. doi:](http://paperpile.com/b/0nGvWM/3vSSI)[10.1371/journal.pcbi.1002801](http://dx.doi.org/10.1371/journal.pcbi.1002801)

30. [Barbu CM, Buttenheim AM, Hancco Pumahuanca ML, Quintanilla Calderón JE, Salazar R, Carrión M, et al. Residual infestation and recolonization during urban Triatoma infestans bug control campaign, Peru. Emerg Infect Dis. 2014;20: 2055–2063.](http://paperpile.com/b/0nGvWM/TFDeU)

31. [Bayer AM, Hunter GC, Gilman RH, Cornejo Del Carpio JG, Naquira C, Bern C, et al. Chagas disease, migration and community settlement patterns in Arequipa, Peru. PLoS Negl Trop Dis. 2009;3: e567.](http://paperpile.com/b/0nGvWM/idTlt)

32. [Bowman NM, Kawai V, Levy MZ, Cornejo del Carpio JG, Cabrera L, Delgado F, et al. Chagas disease transmission in periurban communities of Arequipa, Peru. Clin Infect Dis. 2008;46: 1822–1828.](http://paperpile.com/b/0nGvWM/XweAo)

33. [Buttenheim AM, Paz-Soldan V, Barbu C, Skovira C, Quintanilla Calderón J, Mollesaca Riveros LM, et al. Is participation contagious? Evidence from a household vector control campaign in urban Peru. J Epidemiol Community Health. 2014;68: 103–109.](http://paperpile.com/b/0nGvWM/dj7A2)

34. [Delgado S, Ernst KC, Pumahuanca ML, Yool SR, Comrie AC, Sterling CR, et al. A country bug in the city: urban infestation by the Chagas disease vector *Triatoma infestans* in Arequipa, Peru. Int J Health Geogr. 2013;12: 48.](http://paperpile.com/b/0nGvWM/H9cuM)

35. [Levy MZ, Kawai V, Bowman NM, Waller L a., Cabrera L, Pinedo-Cancino VV, et al. Targeted screening strategies to detect *Trypanosoma cruzi* infection in children. PLoS Negl Trop Dis. 2007;1: e103.](http://paperpile.com/b/0nGvWM/u4Vcx)

36. [Levy MZ, Small DS, Vilhena DA, Bowman NM, Kawai V, Cornejo del Carpio JG, et al. Retracing Micro-Epidemics of Chagas Disease Using Epicenter Regression. PLoS Comput Biol. 2011;7. doi:](http://paperpile.com/b/0nGvWM/HZol7)[10.1371/journal.pcbi.1002146](http://dx.doi.org/10.1371/journal.pcbi.1002146)

37. [Paz-Soldán VA, Bauer KM, Hunter GC, Castillo-Neyra R, Arriola VD, Rivera-Lanas D, et al. To spray or not to spray? Understanding participation in an indoor residual spray campaign in Arequipa, Peru. Glob Public Health. 2016;1692: 1–18.](http://paperpile.com/b/0nGvWM/NwOSK)

38. [Banks K, McDonald SM, Scialom F. Mobile Technology and the Last Mile: “Reluctant Innovation” and FrontlineSMS. Innovations: Technology, Governance, Globalization. MIT Press; 2011;6: 7–12.](http://paperpile.com/b/0nGvWM/ql1VT)

39. [Banks K. FrontlineSMS-A Text Messaging System for NGOs. Oryx. 2007;40: 17.](http://paperpile.com/b/0nGvWM/ppdne)

40. [Banks K, Hersman E. FrontlineSMS and Ushahidi - a demo. 2009 International Conference on Information and Communication Technologies and Development (ICTD). 2009. pp. 484–484.](http://paperpile.com/b/0nGvWM/12o7Q)

41. [Malawi U. RapidSMS Technology. Retrieved June. 2009;](http://paperpile.com/b/0nGvWM/NiJIx)

42. [Blaschke S. An assessment of the use of RapidSMS to monitor the Hepatitis-E outbreak in northern uganda.](http://paperpile.com/b/0nGvWM/QSqUS)

43. [UNICEF’s U-Report: using mobile technology for youth participation in policymaking. In: Center for Public Impact [Internet]. 22 Mar 2016 [cited 22 Feb 2018]. Available:](http://paperpile.com/b/0nGvWM/6f4Yu) <https://www.centreforpublicimpact.org/case-study/unicef-ureport/>

44. [U-Report [Internet]. [cited 22 Feb 2018]. Available:](http://paperpile.com/b/0nGvWM/qXNuZ) <http://ureport.ug/>

45. [Okolloh O. Ushahidi, or “testimony”: Web 2.0 tools for crowdsourcing crisis information. Participatory learning and action. IIED-Participatory Learning and Action; 2009;59: 65–70.](http://paperpile.com/b/0nGvWM/xnbqG)

46. [Puleio M, Jennings V. CycleTel in India: From Proof-of-Concept to Deployment. Presentation to mHealth Working Group. 2011; Available:](http://paperpile.com/b/0nGvWM/BWgAR) <http://coregroup.secure.nonprofitsoapbox.com/storage/CycleTel_mHealth_WG_Jan2011-1.pdf>

47. [Rai RK. Tracking women and children in a Continuum of Reproductive, Maternal, Newborn, and Child Healthcare (RMNCH) in India. J Epidemiol Glob Health. 2014;4: 239–243.](http://paperpile.com/b/0nGvWM/R7pYk)

48. [Lian L. GeoChat on iPhone : A Map-based Chat Application on iPhone [Internet]. 2010. Available:](http://paperpile.com/b/0nGvWM/kO79c) <http://www.diva-portal.org/smash/record.jsf?pid=diva2:351488>

49. [Yang C, Yang J, Luo X, Gong P. Use of mobile phones in an emergency reporting system for infectious disease surveillance after the Sichuan earthquake in China. Bull World Health Organ. 2009;87: 619–623.](http://paperpile.com/b/0nGvWM/3OIRz)

50. [Déglise C, Suzanne Suggs L, Odermatt P. SMS for disease control in developing countries: A systematic review of mobile health applications. J Telemed Telecare. 2012;18: 273–281.](http://paperpile.com/b/0nGvWM/MjU32)

51. [Lewis SL, Feighner BH, Loschen WA, Wojcik RA, Skora JF, Coberly JS, et al. Sages: A suite of freely-available software tools for electronic disease surveillance in resource-limited settings. PLoS One. 2011;6: 1–7.](http://paperpile.com/b/0nGvWM/o5zRj)

52. [Hartung C, Lerer A, Anokwa Y, Tseng C, Brunette W, Borriello G. Open Data Kit: Tools to Build Information Services for Developing Regions. Proceedings of the 4th ACM/IEEE International Conference on Information and Communication Technologies and Development. New York, NY, USA: ACM; 2010. pp. 18:1–18:12.](http://paperpile.com/b/0nGvWM/tv60A)

53. [Tom-Aba D, Olaleye A, Olayinka AT, Nguku P, Waziri N, Adewuyi P, et al. Innovative Technological Approach to Ebola Virus Disease Outbreak Response in Nigeria Using the Open Data Kit and Form Hub Technology. PLoS One. 2015;10: e0131000.](http://paperpile.com/b/0nGvWM/6hrJa)

54. [Mwabukusi M, Karimuribo ED, Rweyemamu MM, Beda E. Mobile technologies for disease surveillance in humans and animals. Onderstepoort J Vet Res. 2014;81: 1–5.](http://paperpile.com/b/0nGvWM/CzFtI)

55. [Aanensen DM, Huntley DM, Feil EJ, Al-Own F ’a, Spratt BG. EpiCollect: Linking smartphones to web applications for epidemiology, ecology and community data collection. PLoS One. 2009;4. doi:](http://paperpile.com/b/0nGvWM/Fyam7)[10.1371/journal.pone.0006968](http://dx.doi.org/10.1371/journal.pone.0006968)

56. [Karimuribo ED, Sayalel K, Beda E, Short N, Wambura P, Mboera LG, et al. Towards One Health disease surveillance: The Southern African Centre for Infectious Disease Surveillance approach. Onderstepoort J Vet Res. 2012;79: 1–7.](http://paperpile.com/b/0nGvWM/p0u8z)

57. [Tumwebaze H, Tumwesigye E, Baeten JM, Kurth AE, Revall J, Murnane PM, et al. Household-Based HIV Counseling and Testing as a Platform for Referral to HIV Care and Medical Male Circumcision in Uganda: A Pilot Evaluation. PLoS One. 2012;7. doi:](http://paperpile.com/b/0nGvWM/f3SAl)[10.1371/journal.pone.0051620](http://dx.doi.org/10.1371/journal.pone.0051620)

58. [Bollinger RC, McKenzie-White J, Gupta A. Building a global health education network for clinical care and research. The benefits and challenges of distance learning tools. Lessons learned from the Hopkins Center for Clinical Global Health Education. Infect Dis Clin North Am. 2011;25: 385–398.](http://paperpile.com/b/0nGvWM/QjatP)

59. [Medic Mobile [Internet]. [cited 22 Feb 2018]. Available:](http://paperpile.com/b/0nGvWM/eX2Nb) <https://medicmobile.org/>

60. [Magpi [Internet]. [cited 22 Feb 2018]. Available:](http://paperpile.com/b/0nGvWM/EZRXn) <https://home.magpi.com/>

61. [Blas MM, Brown B, Menacho L, Alva IE, Silva-Santisteban A, Carcamo C. HPV Prevalence in Multiple Anatomical Sites among Men Who Have Sex with Men in Peru. PLoS One. 2015;10: e0139524.](http://paperpile.com/b/0nGvWM/zDzOG)

62. [Mahmud MA, Spigt M, Mulugeta Bezabih A, López Pavon I, Dinant G-J, Blanco Velasco R. Risk factors for intestinal parasitosis, anaemia, and malnutrition among school children in Ethiopia. Pathog Glob Health. 2013;107: 58–65.](http://paperpile.com/b/0nGvWM/nNkhK)

63. [Human Network International. DataWinners: Data Collection for Development [Internet]. [cited 22 Feb 2018]. Available:](http://paperpile.com/b/0nGvWM/dQccU) <https://www.datawinners.com/>

64. [WebFirst. PhiCollect [Internet]. [cited 22 Feb 2018]. Available:](http://paperpile.com/b/0nGvWM/APF2k) <https://www.webfirst.com/phicollect>

65. [Mtema Z, Changalucha J, Cleaveland S, Elias M, Ferguson HM, Halliday JEB, et al. Mobile Phones As Surveillance Tools: Implementing and Evaluating a Large-Scale Intersectoral Surveillance System for Rabies in Tanzania. PLoS Med. 2016;13: 1–12.](http://paperpile.com/b/0nGvWM/6AdkD)

66. [Haque F, Ball RL, Khatun S, Ahmed M, Kache S, Chisti MJ, et al. Evaluation of a Smartphone Decision-Support Tool for Diarrheal Disease Management in a Resource-Limited Setting. PLoS Negl Trop Dis. 2017;11: 1–19.](http://paperpile.com/b/0nGvWM/LByHa)

67. [Robertson C, Sawford K, Daniel SLA, Nelson TA, Stephen C. Mobile phone-based infectious disease surveillance system, Sri Lanka. Emerg Infect Dis. 2010;16: 1524–1531.](http://paperpile.com/b/0nGvWM/apG8P)

68. [Garcia Sylim P, Cristina S-AC. Development of a low-cost electronic data collection tool for a health facility survey study: lessons learned in the field. Journal of the international society for telemedicine and ehealth. 2016;4: 1–8.](http://paperpile.com/b/0nGvWM/b0pql)

69. [GIS Cloud Mobile Data Collection [Internet]. [cited 22 Feb 2018]. Available:](http://paperpile.com/b/0nGvWM/MlHvF) <https://www.giscloud.com/apps/mobile-data-collection>

70. [Coloma J, Suazo H, Harris E, Holston J. Dengue chat: A novel web and cellphone application promotes community-based mosquito vector control. Annals of global health. 2016;82: 451.](http://paperpile.com/b/0nGvWM/IoEum)

71. [Lozano-Fuentes S, Wedyan F, Hernandez-Garcia E, Sadhu D, Ghosh S, Bieman JM, et al. Cell phone-based system (Chaak) for surveillance of immatures of dengue virus mosquito vectors. J Med Entomol. 2013;50: 879–889.](http://paperpile.com/b/0nGvWM/h78UB)

72. [Bolivia: SMS contra el Chagas. In: Medicos Sin Fronteras [Internet]. [cited 22 Feb 2018]. Available:](http://paperpile.com/b/0nGvWM/jpXHQ) <https://www.msf.es/actualidad/bolivia/bolivia-sms-chagas>

73. [Kimaro HC, Nhampossa JL. Analyzing the problem of unsustainable health information systems in less-developed economies: Case studies from Tanzania and Mozambique. Information Technology for Development. 2005;11: 273–298.](http://paperpile.com/b/0nGvWM/mhNJa)

74. [Brewer CA, Hatchard GW, Harrower MA. ColorBrewer in print: A catalog of color schemes for maps. Cartogr Geogr Inf Sci. 2003;30: 5–32.](http://paperpile.com/b/0nGvWM/w3Sku)

75. [Brewer CA, MacEachren AM, Pickle LW, Herrmann D. Mapping mortality: Evaluating color schemes for choropleth maps. Ann Assoc Am Geogr. 1997;87: 411–438.](http://paperpile.com/b/0nGvWM/pTpX9)

76. [Brewer CA, Pickle L. Evalutation of methods for classifying Epidemiological data on choropleth maps in series. Ann Assoc Am Geogr. 2002;92: 662–681.](http://paperpile.com/b/0nGvWM/L8dZm)

77. [Smith-Jackson TL, Wogalter MS. Users’ Hazard Perceptions of Warning Components: An Examination of Colors and Symbols. Proc Hum Fact Ergon Soc Annu Meet. SAGE Publications Inc; 2000;44: 6–55–6–58.](http://paperpile.com/b/0nGvWM/59roY)

78. [Rasmussen CE, Williams CKI. Gaussian Processes for Machine Learning. MIT Press; 2006.](http://paperpile.com/b/0nGvWM/aD3Mk)

79. [Lindgren F, Rue H, Lindström J. An explicit link between Gaussian fields and Gaussian Markov random fields: the stochastic partial differential equation approach. J R Stat Soc Series B Stat Methodol. 2011;73: 423–498.](http://paperpile.com/b/0nGvWM/VYV5s)

80. [Song H-R, Fuentes M, Ghosh S. A comparative study of Gaussian geostatistical models and Gaussian Markov random field models1. J Multivar Anal. 2008;99: 1681–1697.](http://paperpile.com/b/0nGvWM/6TSOw)

81. [Lindgren F, Rue H. Bayesian Spatial Modelling with R - INLA. J Stat Softw. 2015;63. doi:](http://paperpile.com/b/0nGvWM/j9FN4)[10.18637/jss.v063.i19](http://dx.doi.org/10.18637/jss.v063.i19)

82. [R.Core.Team. R: A language and environment for statistical computing. Vienna, Austria: R Foundation for Statistical Computing; 2017.](http://paperpile.com/b/0nGvWM/SPwCk)

83. [Rose EB, Lee K, Roy JA, Small D, Ross ME, Castillo-Neyra R, et al. Risk maps for cities: Incorporating streets into geostatistical models. Spat Spatiotemporal Epidemiol. 2018; doi:](http://paperpile.com/b/0nGvWM/56VUa)[10.1016/j.sste.2018.08.003](http://dx.doi.org/10.1016/j.sste.2018.08.003)

84. [Ooms J, James D, DebRoy S, Wickham H, Horner J. RMySQL: Database Interface and “MySQL” Driver for R. 2017.](http://paperpile.com/b/0nGvWM/VU2JQ)

85. [Chang W, Cheng J, Allaire JJ, Xie Y, McPherson J. shiny: Web Application Framework for R [Internet]. 2017. Available:](http://paperpile.com/b/0nGvWM/Se3dv) <https://cran.r-project.org/package=shiny>

86. [Cheng J, Karambelkar B, Xie Y. leaflet: Create Interactive Web Maps with the JavaScript “Leaflet” Library [Internet]. 2017. Available:](http://paperpile.com/b/0nGvWM/0iZei) <https://cran.r-project.org/package=leaflet>

87. [Schwarz G. Estimating the Dimension of a Model. Ann Stat. Institute of Mathematical Statistics; 1978;6: 461–464.](http://paperpile.com/b/0nGvWM/XTSG)

88. [Bates D, Mächler M, Bolker B, Walker S. Fitting Linear Mixed-Effects Models Using lme4. Journal of Statistical Software, Articles. 2015;67: 1–48.](http://paperpile.com/b/0nGvWM/Oh3eu)

89. [Geneva: World Health Organization. The MAPS toolkit: mHealth Assessment and Planning for Scale. 2015.](http://paperpile.com/b/0nGvWM/J0Ipg)

90. [Becker S, Miron-Shatz T, Schumacher N, Krocza J, Diamantidis C, Albrecht U-V. mHealth 2.0: Experiences, Possibilities, and Perspectives. JMIR mHealth and uHealth. 2014;2: e24.](http://paperpile.com/b/0nGvWM/wTpCw)

91. [Lescano AG, Larasati RP, Sedyaningsih ER, Bounlu K, Araujo-Castillo RV, Munayco-Escate CV, et al. Statistical analyses in disease surveillance systems. BMC Proc. 2008;2: S7.](http://paperpile.com/b/0nGvWM/MCxLK)

92. [Behrman JR, Gallardo-García J, Parker SW, Todd PE, Vélez-Grajales V. Are Conditional Cash Transfers Effective in Urban Areas? Evidence from Mexico. Educ Econ. 2012;20: 233–259.](http://paperpile.com/b/0nGvWM/ewXAF)

93. [Van Herck P, De Smedt D, Annemans L, Remmen R, Rosenthal MB, Sermeus W. Systematic review: Effects, design choices, and context of pay-for-performance in health care. BMC Health Serv Res. 2010;10: 247.](http://paperpile.com/b/0nGvWM/1CX5X)

94. [Emanuel EJ, Ubel PA, Kessler JB, Meyer G, Muller RW, Navathe AS, et al. Using Behavioral Economics to Design Physician Incentives That Deliver High-Value Care. Ann Intern Med. 2016;164: 114–119.](http://paperpile.com/b/0nGvWM/FN1Y5)

95. [Garard DL, Lippert L, Hunt SK, Paynton ST. Alternatives to traditional instruction: Using games and simulations to increase student learning and motivation. Commun Res Rep. Routledge; 1998;15: 36–44.](http://paperpile.com/b/0nGvWM/vXpAU)

96. [Clark AM, Clark MTG. Pokémon Go and Research: Qualitative, Mixed Methods Research, and the Supercomplexity of Interventions. International Journal of Qualitative Methods. SAGE Publications Inc; 2016;15: 1609406916667765.](http://paperpile.com/b/0nGvWM/Ip1VP)

97. [The World Bank Group. Mobile cellular subscriptions (per 100 people) | Data [Internet]. [cited 26 Feb 2018]. Available:](http://paperpile.com/b/0nGvWM/DmqoS) <https://data.worldbank.org/indicator/IT.CEL.SETS.P2?end=2016&start=2016&view=map>

98. [Gozzer Infante E. Una visión panorámica de las experiencias de telesalud en Perú. Rev Peru Med Exp Salud Publica. 2015;32: 385–390.](http://paperpile.com/b/0nGvWM/k0Obo)

99. [Soto G, Araujo-Castillo RV, Neyra J, Fernandez M, Leturia C, Mundaca CC, et al. Challenges in the implementation of an electronic surveillance system in a resource-limited setting: Alerta, in Peru. BMC Proc. 2008;2 Suppl 3: S4.](http://paperpile.com/b/0nGvWM/DFtfW)

100. [García PJ, Vargas JH, Caballero PN, Calle JV, Bayer AM. An e-health driven laboratory information system to support HIV treatment in Peru: E-quity for laboratory personnel, health providers and people living with HIV. BMC Med Inform Decis Mak. 2009;9: 1–11.](http://paperpile.com/b/0nGvWM/PeWbJ)

101. [Leal-Neto OB, Dimech GS, Libel M, Oliveira W, Ferreira JP. Digital disease detection and participatory surveillance: overview and perspectives for Brazil. Rev Saude Publica. 2016;50: 17.](http://paperpile.com/b/0nGvWM/LR8uH)

102. [Delgado S, Neyra RC, Machaca VRQ, Juárez JA, Chu LC, Verastegui MR, et al. A history of Chagas disease transmission, control, and re-emergence in peri-rural La Joya, Peru. PLoS Negl Trop Dis. 2011;5. doi:](http://paperpile.com/b/0nGvWM/MnSGo)[10.1371/journal.pntd.0000970](http://dx.doi.org/10.1371/journal.pntd.0000970)

**Leyendas de la información de soporte**

**S1. Datos del estudio.** Datos colectados en el studio comparando la vigilancia con la aplicación VectorPoint y con mapas de papel.

**S2. Articulo traducido al Español.**
